# Anti-Inflammatory and Antimicrobial Activities of Extracts Developed from *Symphytum officinale* Leaves, Flowers, and Roots: Experimental Evaluation and Molecular Docking Study

**DOI:** 10.3390/ijms27188060

**Published:** 2026-09-10

**Authors:** Oleh Koshovyi, Getter Dolgošev, Andrii Kaplaushenko, Oleksandr Panasenko, Volodymyr Zazharskyi, Yuriy Karpenko, Roman Shcherbyna, Andriy Hotsulia, Mariia Shanaida, Ivo Laidmäe, Jyrki Heinämäki, Ain Raal

**Affiliations:** 1 Institute of Pharmacy, Faculty of Medicine, University of Tartu, 50411 Tartu, Estonia; getter.dolgosev@ut.ee (G.D.); ivo.laidmae@ut.ee (I.L.); jyrki.heinamaki@ut.ee (J.H.); ain.raal@ut.ee (A.R.); 2 Zaporizhzhia State Medical and Pharmaceutical University, 69035 Zaporizhzhia, Ukraine; kaplaushenko.a.g@zsmu.edu.ua (A.K.); panasenko.o.i@zsmu.edu.ua (O.P.); karpenko.y.v@zsmu.edu.ua (Y.K.); shcherbyna.r.o@zsmu.edu.ua (R.S.); hotsulia.a.s@zsmu.edu.ua (A.H.); 3 Dnipro State Agrarian and Economic University, 49009 Dnipro, Ukraine; zazharskyi.v.v@dsau.dp.ua; 4 I. Horbachevsky Ternopil National Medical University, 46001 Ternopil, Ukraine; shanayda@tdmu.edu.ua

**Keywords:** *Symphytum officinale* L., Boraginaceae, comfrey, extract, anti-inflammatory activity, antimicrobial activity, molecular docking

## Abstract

*Symphytum officinale* L. is a traditional medicinal plant widely used for wound healing and bone regeneration; however, comparative studies evaluating the biological activities of extracts prepared from different plant organs remain limited. The present study compared the antimicrobial and anti-inflammatory activities of organ-specific *S. officinale* extracts and investigated their potential molecular mechanisms of bioactivities using molecular docking. Antimicrobial activity was evaluated against six bacterial reference strains by broth serial dilution, whereas anti-inflammatory activity was assessed in a serotonin-induced paw oedema model in rats together with the determination of serum prostaglandin E_2_ (PGE_2_), tumour necrosis factor-α (TNF-α), and nitrotyrosine levels. Molecular docking was performed against bacterial peptide deformylases, cyclooxygenase-2 (COX-2), and 5-lipoxygenase (5-LOX). The 40% ethanolic leaf extract (S-7) and 70% ethanolic flower extract (S-13) exhibited the broadest antimicrobial spectrum, with pronounced activity against *S. aureus*, *E. faecalis*, *E. coli*, and *L. monocytogenes*. The 70% ethanolic leaf extract (S-8) demonstrated the strongest early anti-exudative activity (47.50%), whereas the 70% ethanolic flower extract (S-13) most effectively reduced PGE_2_, TNF-α, and nitrotyrosine levels. Molecular docking suggested that kaempferol-3-O-glucoside may be the principal contributor to antimicrobial activity through interactions with bacterial peptide deformylases, while hyperoside, isoquercitrin, and rosmarinic acid exhibited favourable interactions with COX-2 and 5-LOX, indicating their key role in exerting anti-inflammatory effects. These findings demonstrate pronounced organ-specific pharmacological differences among *S. officinale* extracts and identify hydroethanolic leaf, flower, and root extracts as promising candidates for further development as multitarget wound-healing phytopharmaceuticals.

## 1. Introduction

*Symphytum officinale* L. (Boraginaceae), commonly known as comfrey, has been widely used in traditional medicine for centuries to support wound and fracture healing and to relieve musculoskeletal pain, including disorders affecting muscles and joints [1,2].

The roots of *S. officinale* are the plant part most frequently used in medicinal preparations because they are rich in allantoin, a bioactive compound known to promote tissue regeneration and wound repair. Although leaves contain substantially lower concentrations of allantoin, they have also demonstrated wound-healing activity, which has been attributed to their phenolic compounds, polysaccharide fraction and other bioactive molecules [3,4]. In addition to roots and leaves, the aerial parts of *S. officinale* have been used in traditional medicine; however, their pharmacological potential has received considerably less scientific attention. Recently, we reported the first comprehensive comparison of the organ-specific phytochemical profiles of *S. officinale* roots, leaves, and flowers, demonstrating marked differences in the distribution of phenolic compounds and pyrrolizidine alkaloids among these plant organs [5]. The principal phenolic constituents identified included kaempferol-3-O-glucoside, hyperoside, isoquercitrin, rosmarinic acid, caffeic acid, and ferulic acid, whereas intermedine, lycopsamine, and their corresponding N-oxides represented the major pyrrolizidine alkaloids. Considering the pronounced organ-specific differences in phytochemical composition previously demonstrated for *S. officinale*, it is reasonable to hypothesise that extracts prepared from different plant organs possess distinct antimicrobial and anti-inflammatory activities.

The therapeutic application of *S. officinale* is substantially limited by the presence of 1,2-unsaturated pyrrolizidine alkaloids (PAs), including intermedine, lycopsamine, and their N-oxides [5,6,7], which may undergo metabolic activation to reactive pyrrolic metabolites capable of inducing hepatotoxicity, genotoxicity, and carcinogenicity. Oral exposure to PA-containing preparations has been associated with hepatic sinusoidal obstruction syndrome (previously known as hepatic veno-occlusive disease), and regulatory authorities therefore recommend avoiding the internal use of comfrey preparations. In contrast, topical preparations containing controlled amounts of PAs may be used for limited periods under appropriate regulatory restrictions. These safety considerations emphasize the importance of developing standardized extracts with reduced PA content while preserving their therapeutic efficacy [8,9,10].

The pharmacological properties of *S. officinale* have been investigated in a variety of experimental and clinical settings. The study of *Symphytum officinale* root extract showed a significant beneficial effect on human skin fibroblasts, promoting wound healing [11]. Due to the presence of hepatotoxic pyrrolizidine alkaloids, the internal use of *S. officinale* crude extracts is limited [12]. However, topical application may present a lower systemic risk because of the relatively limited dermal absorption of PAs, provided that residual PA levels and duration of use are appropriately controlled [8,9]. In addition to its well-established wound-healing activity, *S. officinale* has demonstrated beneficial effects on bone regeneration, including increased bone mineral density and enhanced osteogenesis [13,14]. Root-derived topical preparations have been reported to accelerate wound healing and exhibit antimicrobial activity [15], whereas leaf extracts have been shown to promote tissue repair by reducing inflammation and stimulating collagen deposition [16]. Clinical studies have further confirmed the efficacy of *S. officinale* preparations in relieving pain and reducing oedema associated with ankle sprains [17]. Beyond these traditional indications, extracts of *S. officinale* have also shown α-glucosidase inhibitory activity, suggesting potential applications in the management of type 2 diabetes mellitus [18]. Collectively, these findings highlight the broad pharmacological potential of *S. officinale* and support its continued investigation as a source of bioactive phytochemicals. More broadly, contemporary studies of medicinal plant extracts increasingly integrate advanced chemical profiling, such as LC–MS-based approaches, with functional pharmacological assays and complementary computational analyses to investigate relationships between phytochemical composition and biological activity [19,20]. Such integrated approaches are particularly relevant for chemically complex plant preparations, in which multiple constituents may contribute to anti-inflammatory effects. Despite the available evidence supporting the pharmacological potential of *S. officinale*, the relationship between plant organ-specific phytochemical composition and biological activity remains insufficiently understood. Previous studies have primarily focused on individual plant organs, particularly roots or leaves, whereas direct comparative evaluations of root, leaf, and flower extracts prepared under comparable extraction conditions are scarce. Moreover, it remains unclear whether the marked organ-specific differences in phenolic composition previously demonstrated by our group are reflected in differences in antimicrobial and anti-inflammatory activities. Establishing such relationships is important for the rational selection of the most pharmacologically promising plant organ and extraction conditions for further development of *S. officinale*-derived preparations. Therefore, building upon our previous phytochemical characterization of *S. officinale* root, leaf, and flower extracts [5], the present study aimed to comparatively evaluate their antimicrobial and anti-inflammatory activities and to investigate potential molecular mechanisms underlying these effects using molecular docking [21,22]. Specifically, we sought to identify the most biologically active organ-specific extracts and to relate the experimentally observed activities to their previously established phytochemical profiles and predicted interactions of major constituents with bacterial peptide deformylases and inflammation-related targets, including COX-2 and 5-LOX.

**Figure 1:**
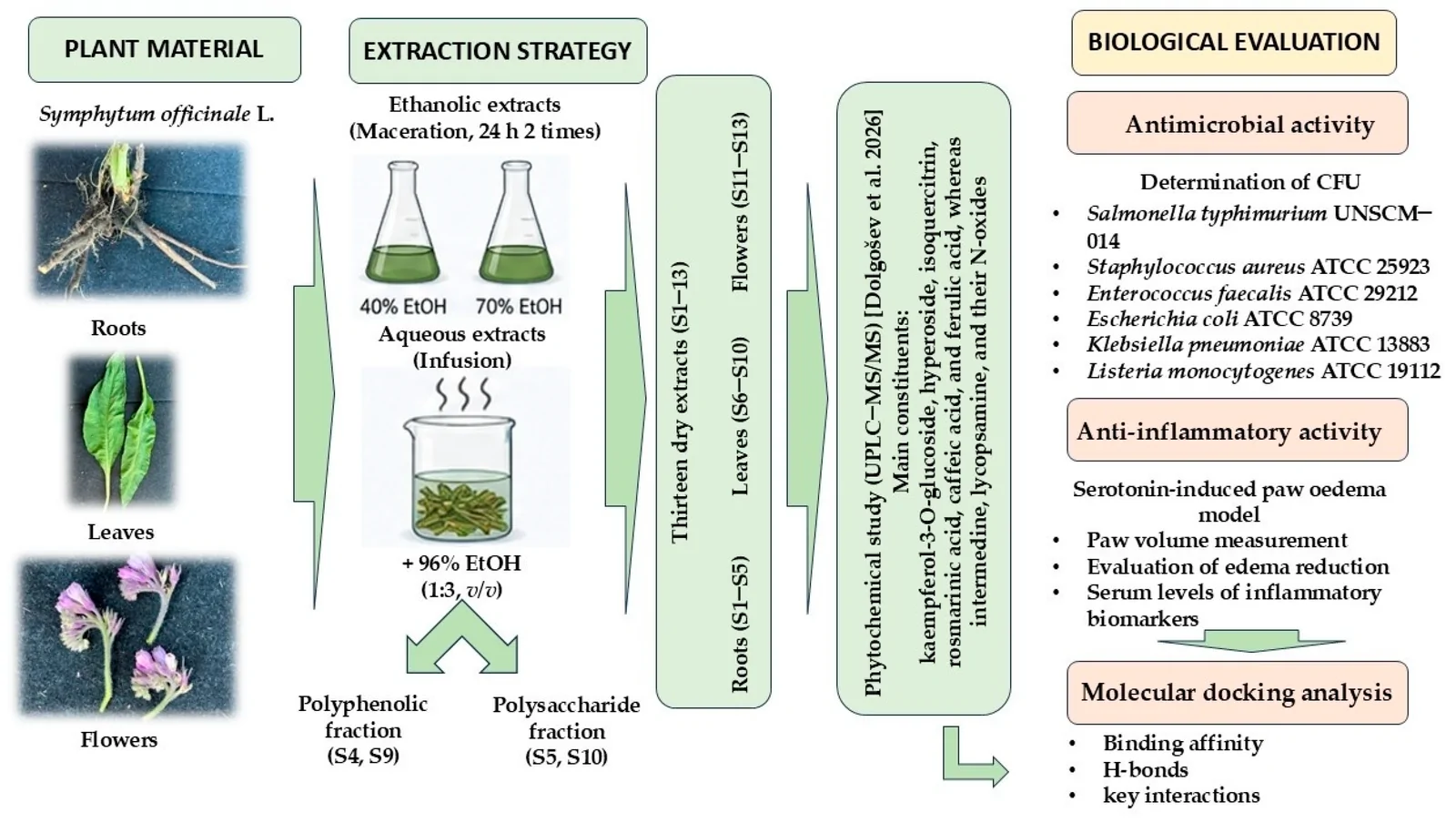
Schematic overview of the study design, including the preparation of *Symphytum officinale* extracts, biological evaluation, and molecular docking analysis.

A schematic overview of the experimental design, including extract preparation, biological evaluation, and molecular docking analysis, is presented in Figure 1.

## 2. Results

### 2.1. Antimicrobial Activity

To evaluate the antimicrobial potential of the *S. officinale* extracts and their phenolic and polysaccharide fractions, bacterial growth was assessed using a broth serial dilution assay. Quantitative antimicrobial effects were evaluated based on viable bacterial counts expressed as CFU/mL at the tested extract concentrations. For comparative analysis, Table 1 presents bacterial growth at the fourth serial dilution (2 µg/mL), whereas the complete concentration-dependent CFU profiles obtained at 2, 4, 8, and 16 µg/mL for all tested bacterial strains are provided in Figure S1. This presentation enables comparison of the antimicrobial activity of all investigated samples against Gram-positive and Gram-negative bacterial strains while providing the complete concentration-dependent dataset in the Supplementary Materials.

**Figure 2:**
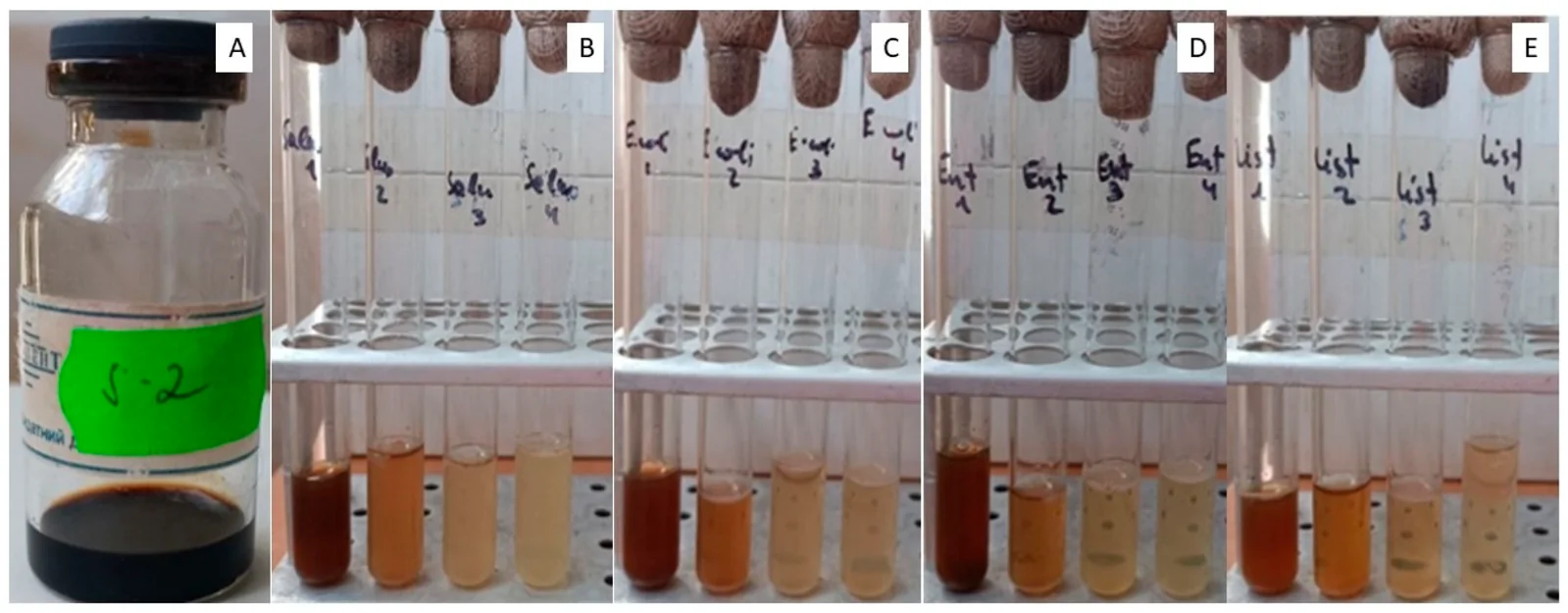
Representative broth serial dilution assay showing bacterial growth in the presence of extract S-2 (**A**) against *S. enterica* (**B**), *E. coli* (**C**), *E. faecalis* (**D**), and *L. monocytogenes* (**E**).

The antimicrobial activity of *S. officinale* extracts was evaluated against six bacterial reference strains using the broth serial dilution assay. Considerable differences in antimicrobial efficacy were observed among the tested extracts, indicating that both extract composition and bacterial species influenced susceptibility. Among the investigated samples, extracts S-2, S-5, S-6, S-7, S-10, and S-13 exhibited the strongest inhibitory activity, whereas S-3, S-4, S-11, and S-12 showed comparatively weak or limited antibacterial effects (Table 1). Gram-positive bacteria were generally more susceptible to the tested extracts than Gram-negative microorganisms. The strongest inhibition was observed against *L. monocytogenes*, *S. aureus*, and *E. faecalis*. Extract S-2 markedly reduced bacterial growth of *L. monocytogenes*, *S. aureus*, and *E. faecalis*, producing low CFU values of 0.63 × 10^9^, 1.16 × 10^9^, and 0.75 × 10^9^ CFU/mL, respectively, at the fourth dilution. According to the predefined CFU-based inhibitory endpoint, S-2 reached the inhibitory threshold at ≤2 µg/mL against *E. faecalis* and *L. monocytogenes* and at 4 µg/mL against *S. aureus* (Figure 2).

**Figure 3:**
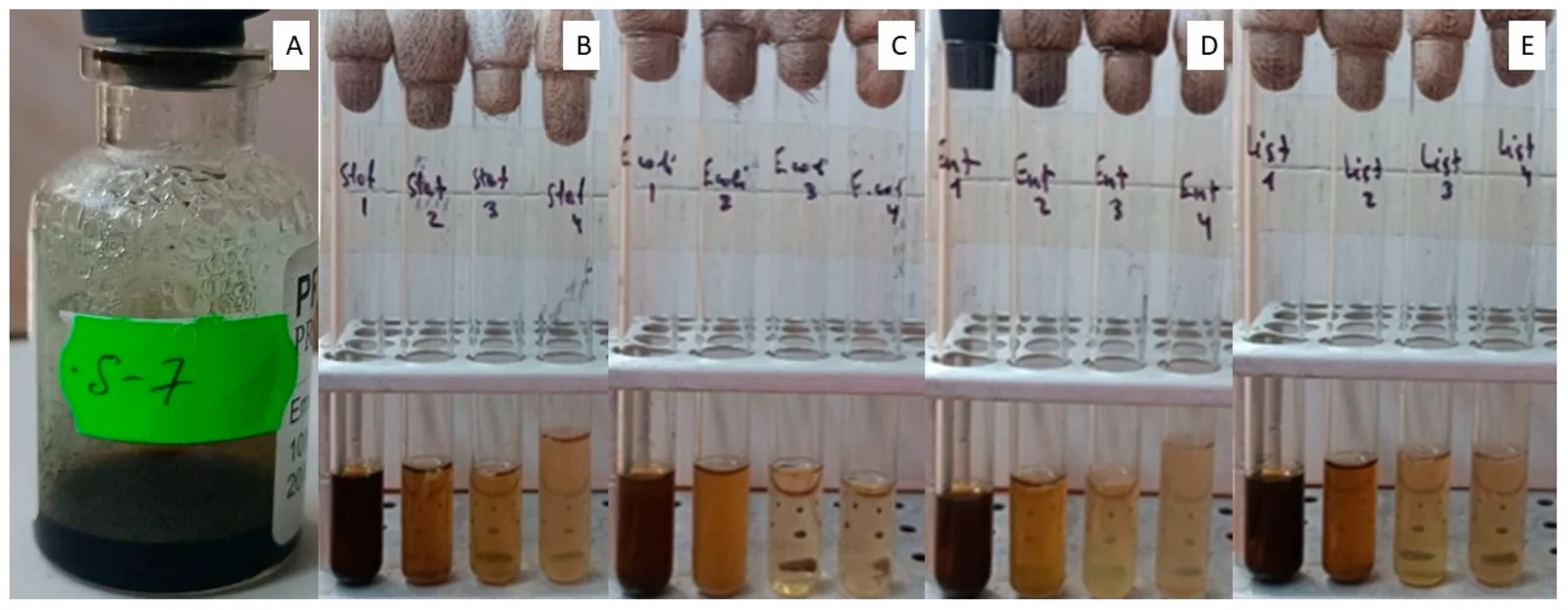
Representative broth serial dilution assay showing bacterial growth in the presence of extract S-7 (**A**) against *S. aureus* (**B**), *E. coli* (**C**), *E. faecalis* (**D**), and *L. monocytogenes* (**E**).

Similar activity was demonstrated by extract S-5, which substantially inhibited all three Gram-positive species. Extract S-6 showed selective activity, with the most pronounced inhibition against *L. monocytogenes*. Extract S-10 effectively suppressed the growth of *L. monocytogenes*, with additional moderate activity against *S. aureus* and *E. faecalis*. Among all investigated samples, extract S-7 demonstrated one of the strongest antibacterial profiles, producing pronounced inhibition of all tested Gram-positive bacteria and *E. coli* (Figure 3).

The inhibitory activity against Gram-negative bacteria was generally lower than that observed for Gram-positive microorganisms. *K. pneumoniae* was the least susceptible species, with most extracts producing only moderate reductions in bacterial growth. Likewise, *S. enterica* serovar Typhimurium exhibited only moderate susceptibility. In contrast, extract S-7 demonstrated substantial activity against *E. coli*, reducing bacterial growth to 0.32 × 10^9^ CFU/mL, representing one of the strongest inhibitory effects observed among Gram-negative bacteria. Extract S-13 showed a similar profile, with marked inhibition of *E. coli* to 0.28 × 10^9^ CFU/mL at the fourth dilution. According to the CFU-based inhibitory endpoint used for comparative analysis, both S-7 and S-13 reached the predefined inhibitory threshold at ≤2 µg/mL against *E. coli*.

Overall, extracts S-2, S-5, S-6, S-7, S-10, and S-13 displayed the highest antimicrobial potential. Extracts S-7 and S-13 exhibited the broadest antibacterial spectrum, showing pronounced activity against *S. aureus*, *E. faecalis*, *E. coli*, and *L. monocytogenes*. In contrast, extracts S-3, S-4, S-11, and S-12 demonstrated weak or limited inhibitory activity, with the predefined CFU-based inhibitory endpoint not being reached at concentrations up to 16 µg/mL for most tested bacterial strains.

**Figure 4:**
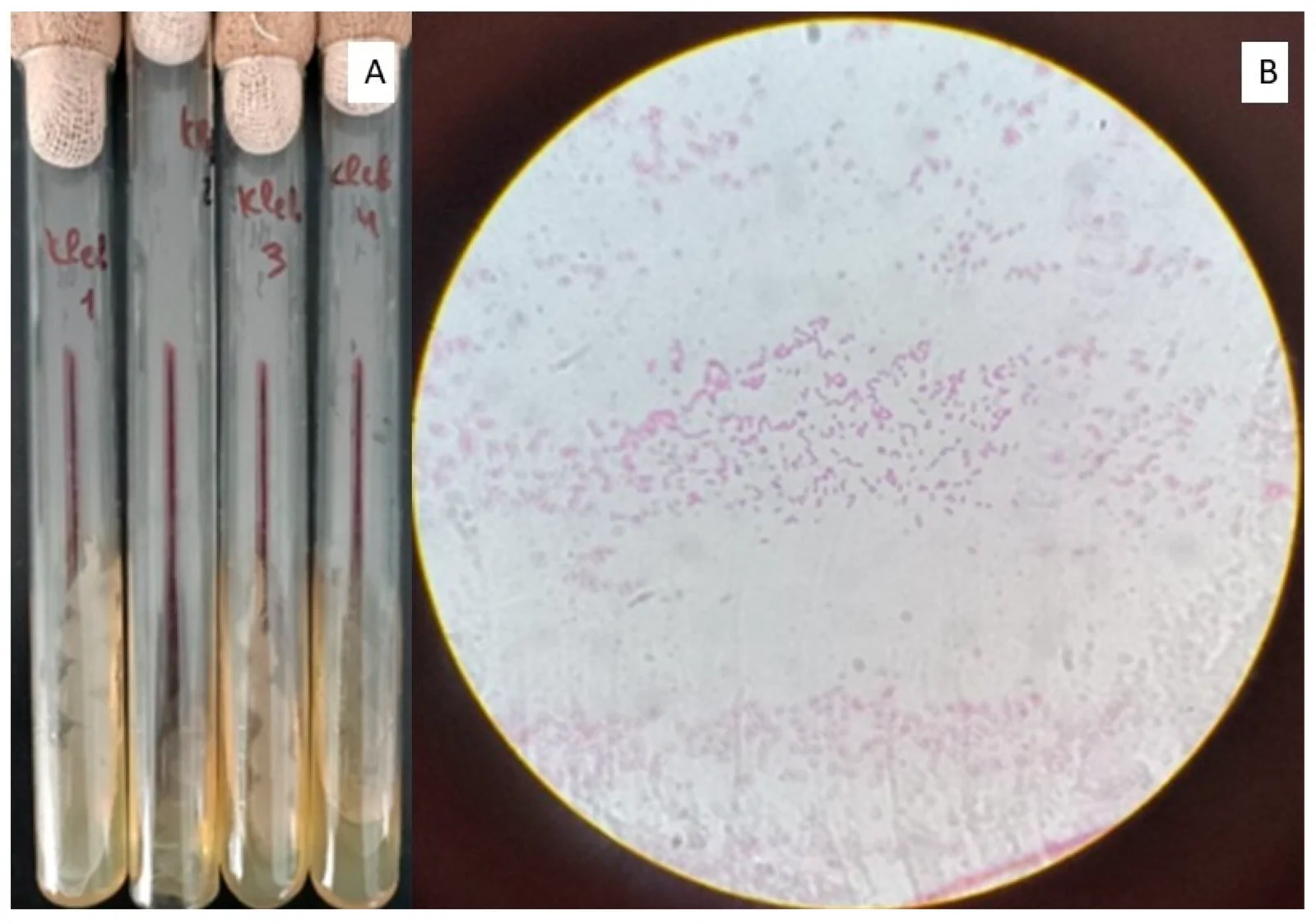
*K. pneumoniae* culture: growth on the medium (**A**) and Gram-stained smear preparations (**B**) ×900.

Representative culture growth and Gram-stained microscopic morphology of *K. pneumoniae* used in the antimicrobial assay are shown in Figure 4.

Overall, the results indicate that the antimicrobial activity of the extracts and polysaccharide fractions of *S. officinale* exhibited clear selectivity and depended on both the nature of the sample and the microorganism species. Based on the overall set of indicators, extracts S-7 and S-13 may be considered the most promising samples, as they demonstrated pronounced inhibitory activity against four of the six tested bacterial strains, including *S. aureus*, *E. faecalis*, *E. coli*, and *L. monocytogenes*. Extracts S-2, S-5, S-6, and S-10 also exhibited selective antimicrobial activity, primarily against Gram-positive bacteria, particularly *S. aureus*, *E. faecalis*, and *L. monocytogenes*. The weakest activity was observed for S-3, S-4, and S-12, for which the inhibitory effect was limited or absent against most tested microorganisms.

### 2.2. Anti-Inflammatory Activity

The serotonin-induced paw oedema model confirmed the development of acute inflammatory oedema following subplantar administration of 0.1 mL of a 0.5% serotonin solution. This experimental model is considered particularly suitable for evaluating compounds with potential anti-serotonergic and anti-inflammatory properties. The initial paw volumes were comparable across all animals, with the control group measuring 1.25 ± 0.17 mL. Following serotonin administration, the inflammatory control group exhibited a progressive increase in paw oedema throughout the experiment, reaching volume increments (ΔV) of 0.88, 1.41, and 1.94 mL at 1, 3, and 5 h, respectively (Table 2).

Among the reference drugs, diclofenac demonstrated pronounced anti-exudative activity, reaching 30.96% inhibition of oedema after 1 h and achieving its maximum effect (39.60%) at 3 h. Quercetin exhibited weaker anti-inflammatory activity, with the highest anti-exudative effect of 26.14% also observed at 3 h.

Among the tested *S. officinale* extracts, extract S-8 showed the highest anti-exudative activity during the early phase of inflammation, producing 47.50% inhibition of oedema at 1 h. Extracts S-11 and S-6 also demonstrated marked activity, with anti-exudative effects of 43.67% and 39.27%, respectively. At 3 h after serotonin administration, extract S-13 exhibited the strongest anti-inflammatory effect, with an anti-exudative activity of 50.85%. Moreover, at the final observation point (5 h), S-13 reduced paw oedema more effectively than diclofenac, producing inhibition rates of 37.32% and 30.66%, respectively (Table 2).

To further characterise the anti-inflammatory activity of the investigated extracts, serum inflammatory biomarkers were quantified after completion of the experiment (Table 3). In the inflammatory control group, all measured biomarkers were markedly elevated compared with control animals. Serum TNF-α increased from 39.93 to 536.71 pg/mL, prostaglandin E_2_ (PGE_2_) increased from 315.61 to 1540.65 pg/mL, and nitrotyrosine increased from 11.43 to 62.81 ng/mL.

Among all tested extracts, S-13 produced the most pronounced reduction in PGE_2_ levels, decreasing its concentration by 76.77%, an effect that was slightly greater than that observed with diclofenac and substantially greater than that observed with quercetin. Furthermore, S-13 markedly reduced serum TNF-α and nitrotyrosine levels by 75.41% and 73.84%, respectively. Extract S-8 also demonstrated substantial anti-inflammatory activity, producing the greatest reductions in TNF-α (75.51%) and nitrotyrosine (74.41%) among the investigated samples (Table 3).

### 2.3. Molecular Docking Analysis of Antimicrobial Targets

Molecular docking of the major phytochemical constituents identified in *S. officinale* extracts [5] was performed against bacterial peptide deformylases from *E. coli* (PDB ID: 1LRU), *S. aureus* (PDB ID: 1Q1Y), and *E. faecalis* (PDB ID: 2OS1), which were selected as molecular targets associated with bacterial viability. Among the investigated compounds, isoquercitrin demonstrated the highest predicted binding affinity toward all three peptide deformylases. The calculated binding energies were −5.6 kcal/mol, −10.9 kcal/mol, and −9.8 kcal/mol for PDB IDs 1LRU, 1Q1Y, and 2OS1, respectively (Table 4).

Hyperoside, isoquercitrin, and rosmarinic acid also exhibited favourable binding affinities toward the investigated bacterial targets, although their predicted binding energies were generally higher than those of kaempferol-3-O-glucoside (Table 4). Analysis of the docking poses revealed that ligand binding was stabilised by multiple non-covalent interactions, including hydrogen bonds, π-interactions, hydrophobic contacts, and electrostatic interactions with amino acid residues located within the active sites of the investigated enzymes (Table 4).

Compared with the reference ligand actinonin, a well-established inhibitor of bacterial peptide deformylase, kaempferol-3-O-glucoside, isoquercitrin, and hyperoside exhibited the greatest similarity in their binding interaction patterns.

**Figure 5:**
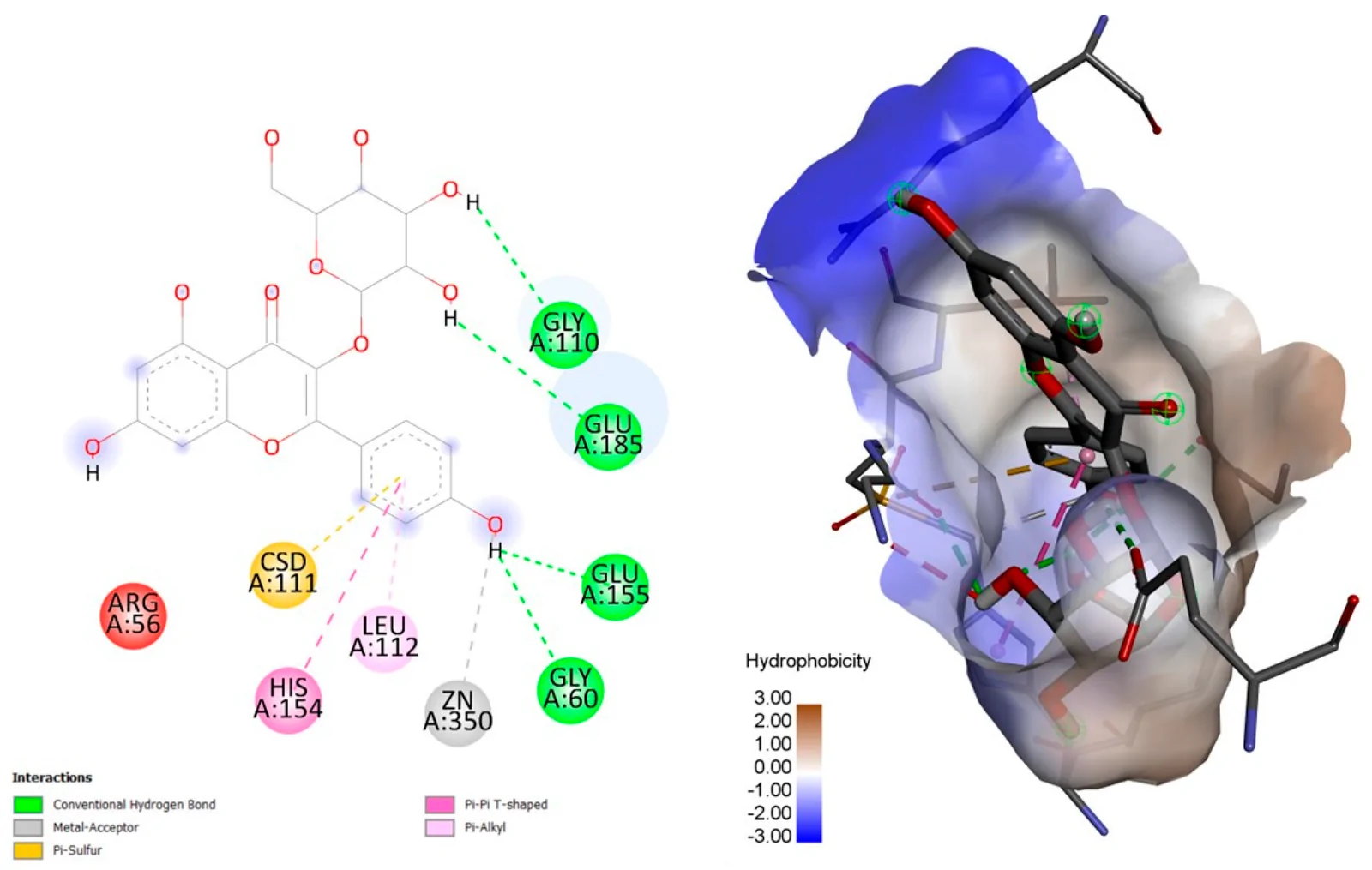
Two-dimensional representation of the docking pose of kaempferol-3-O-glucoside within the active site of *Staphylococcus aureus* peptide deformylase (PDB ID: 1Q1Y).

Particular attention should be paid to kaempferol-3-O-glucoside, which was predicted to form a metal-acceptor interaction with the catalytic Zn^2+^ ion, a key structural feature required for inhibition of the metallo-dependent peptide deformylase. The docking pose revealed that the flavonoid moiety of the ligand was deeply accommodated within the hydrophobic region of the enzyme active site, whereas the glucoside moiety was oriented toward the polar environment of the binding pocket (Figure 5).

Rosmarinic acid also formed stable complexes with the investigated bacterial peptide deformylases, particularly with the *Enterococcus faecalis* enzyme (PDB ID: 2OS1). However, based on the overall docking profile, its predicted binding affinities were generally lower than those of the investigated flavonoid glycosides (Table 4).

Evaluation of the anti-inflammatory potential of the investigated phytochemicals against 5-lipoxygenase (5-LOX; PDB ID: 3O8Y) identified hyperoside as the compound with the highest predicted binding affinity, exhibiting a binding energy of −9.2 kcal/mol. Favourable interactions with 5-LOX were also observed for kaempferol-3-O-glucoside and isoquercitrin, with predicted binding energies of −8.6 and −8.4 kcal/mol, respectively (Table 4).

**Figure 6:**
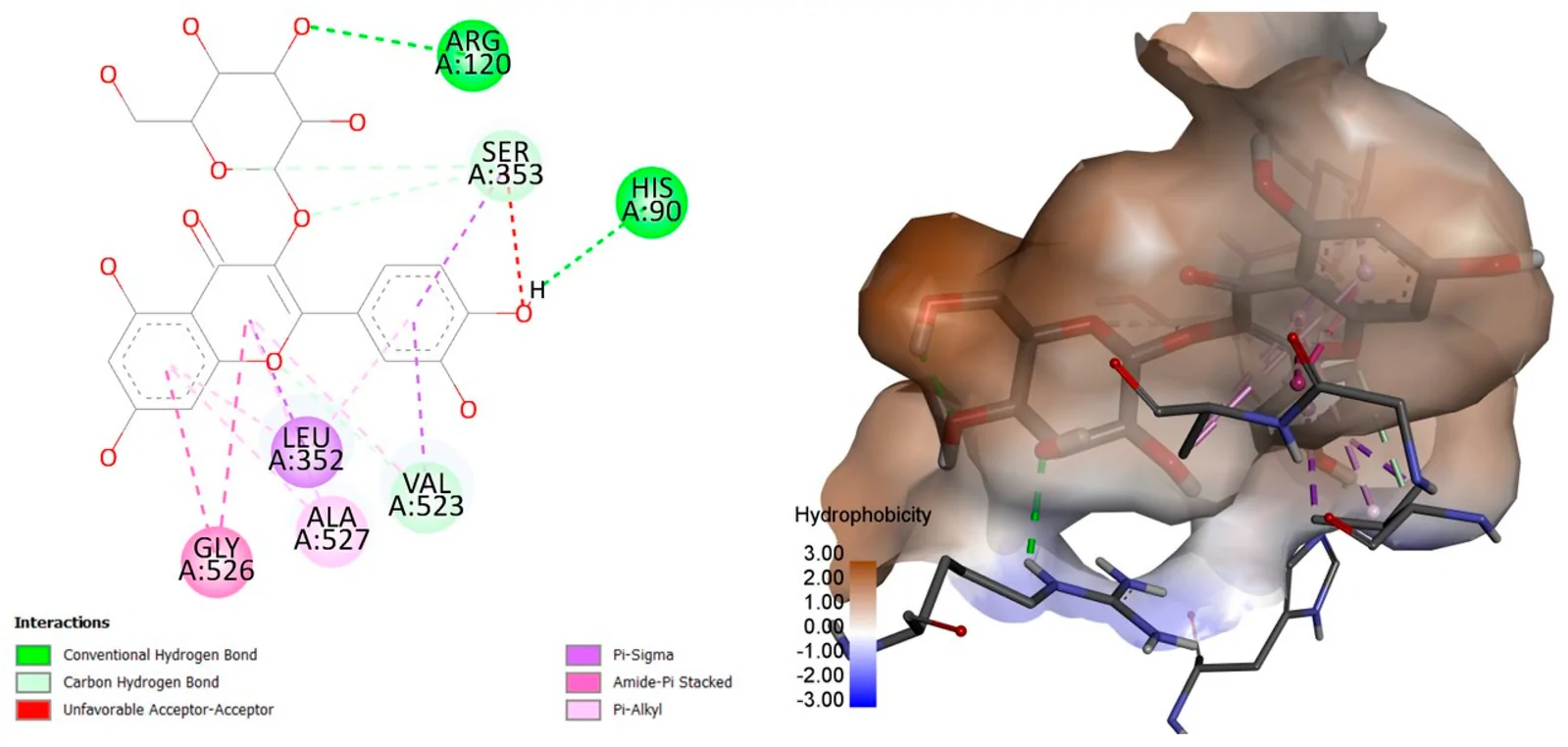
Two-dimensional interaction diagram of hyperoside docked into the active site of cyclooxygenase-2 (COX-2; PDB ID: 5IKR).

Among all investigated compounds, hyperoside demonstrated the strongest predicted affinity toward cyclooxygenase-2 (COX-2; PDB ID: 5IKR), with a calculated binding energy of −10.3 kcal/mol. Analysis of the two-dimensional interaction diagram revealed that stabilisation of the ligand–protein complex was primarily mediated by multiple intermolecular hydrogen bonds (Figure 6). Additional stabilisation was provided by carbon–hydrogen interactions, which contributed to the proper orientation of the ligand within the binding pocket. Furthermore, the aromatic moiety of hyperoside formed several mediated interactions, including π-sigma, amide–π stacked, and π-alkyl contacts, with amino acid residues of the active site, indicating that hydrophobic and π-mediated contacts play an important role in stabilising the ligand–protein complex (Figure 6).

The high predicted binding affinity of hyperoside toward the COX-2 active site may be attributed to the presence of its flavonoid scaffold, which is capable of establishing multiple favourable interactions with amino acid residues within the binding pocket. These interactions include π-mediated contacts involving amino acid residues as well as numerous intermolecular hydrogen bonds formed by the hydroxyl groups of the ligand. The glycosidic moiety further contributes to complex stabilisation by enhancing polar interactions within the active site.

A similar interaction profile with COX-2 was observed for isoquercitrin and rosmarinic acid, both of which exhibited predicted binding energies of −9.5 kcal/mol. Kaempferol-3-O-glucoside showed a slightly lower binding affinity (−8.2 kcal/mol), although its binding pose was likewise stabilised by a combination of hydrophobic contacts and hydrogen-bonding interactions. The remaining phytochemicals demonstrated moderate predicted affinities toward COX-2, suggesting a comparatively smaller contribution to the overall anti-inflammatory potential of the investigated extracts.

## 3. Discussion

The present study demonstrated that Gram-positive bacteria were generally more susceptible to the developed *S. officinale* extracts than Gram-negative microorganisms. This observation is consistent with the well-established structural differences among bacterial cell envelopes. The outer membrane of Gram-negative bacteria restricts the penetration of numerous plant-derived secondary metabolites, including phenolic acids, flavonoids, and tannins, thereby reducing antibacterial efficacy [23].

The observed differences in antimicrobial activity among the extracts most likely reflect variations in their phytochemical composition. In our previous study, significant organ-specific differences in the chemical profiles of *S. officinale* aerial parts and roots were demonstrated, including variations in phenolic compounds and pyrrolizidine alkaloids. The higher antibacterial activity of extracts S-2, S-5, S-6, S-7, S-10, and S-13 may therefore be associated with a greater abundance of bioactive constituents acting either individually or synergistically [3,24].

The antibacterial effects were clearly selective. *Listeria monocytogenes*, *Enterococcus faecalis*, and *Staphylococcus aureus* were consistently among the most susceptible microorganisms, whereas *Klebsiella pneumoniae* and *Salmonella enterica* serovar Typhimurium demonstrated lower susceptibility. Such selectivity has also been reported for extracts rich in phenolic compounds, which may preferentially affect Gram-positive bacteria through disruption of membrane integrity, inhibition of cell-wall-related processes, and interference with essential metabolic enzymes [23,24,25].

Among all investigated samples, extracts S-7 and S-13 deserve particular attention because they exhibited the broadest antibacterial spectrum, effectively inhibiting not only Gram-positive bacteria but also *Escherichia coli*. This broader activity suggests that these samples may contain a more favourable combination of phytochemicals that interact with multiple bacterial targets. In contrast, S-12 showed weak activity against all tested strains, while S-3 and S-4 were also characterised by limited inhibitory effects.

Overall, the antimicrobial activity of *S. officinale* extracts was species-dependent, with S-7 and S-13 exhibiting the broadest antibacterial spectrum. The generally lower susceptibility of Gram-negative bacteria, particularly *K. pneumoniae* and *S. enterica serovar Typhimurium*, may be related to the additional outer membrane permeability barrier, which limits the penetration of many plant-derived bioactive compounds, together with intrinsic resistance mechanisms [16,20]. Nevertheless, the pronounced activity of S-7 and S-13 against *E. coli* indicates that selected extracts may partially overcome this barrier.

To provide an integrated overview of the antimicrobial activity of the investigated extracts and polysaccharide fractions, a heatmap summarising their inhibitory effects against the tested bacterial strains was constructed (Table 5). The visualisation confirms the superior antibacterial profiles of S-7 and S-13 and illustrates the selective susceptibility of the tested microorganisms.

Previous experimental studies demonstrated moderate antibacterial activity of *S. officinale* leaf and root extracts, with the highest activity generally observed for hydroalcoholic preparations, particularly against *S. aureus* [26,27]. Our results are consistent with these observations and confirm the superior activity of hydroethanolic extracts. However, unlike previous studies, we systematically compared 13 extracts and fractions prepared from roots, leaves, and flowers and demonstrated pronounced organ-specific differences in biological activity. The 40% ethanolic leaf extract (S-7) and the 70% ethanolic flower extract (S-13) exhibited the broadest antibacterial spectrum. These differences in activity may be related to their distinct organ-specific phytochemical profiles reported previously [5]. Furthermore, a recent article by Ibrahim et al. demonstrated that *S. officinale* extracts inhibit *Porphyromonas gingivalis* and exhibit synergistic activity with metronidazole, further supporting the broad antimicrobial potential of this species [28]. The present study further extends previous investigations by combining experimental pharmacological evaluation with molecular docking analysis. While earlier reports primarily described antibacterial activity, our docking results provide a plausible molecular explanation by identifying kaempferol-3-O-glucoside, hyperoside, and isoquercitrin as the principal compounds interacting with bacterial peptide deformylases.

The present study demonstrated that *S. officinale* extracts exhibit pronounced anti-inflammatory activity in the serotonin-induced paw oedema model, although substantial differences in efficacy were observed among the samples tested. Among all extracts, S-8 and S-13 exhibited the most pronounced anti-inflammatory effects, suggesting that their phytochemical composition may provide complementary mechanisms contributing to inflammation control. Extract S-8 showed the highest anti-exudative activity during the early phase of inflammation, reaching 47.50% inhibition of paw oedema within the first hour after serotonin administration. Because serotonin-induced oedema primarily reflects the vascular phase of acute inflammation, this finding suggests that S-8 may interfere with serotonin-mediated increases in vascular permeability or modulate the release of vasoactive mediators [29,30]. The greater efficacy of S-8 compared with diclofenac during the first hour further indicates that its pharmacological activity is not restricted to cyclooxygenase inhibition and may involve additional mechanisms acting during the early inflammatory response. In contrast, extract S-13 exhibited its maximum anti-inflammatory effect during the later phase of inflammation. Although its activity developed more gradually, S-13 produced sustained inhibition of paw oedema and remained more effective than diclofenac at the final observation time point. These findings suggest that S-13 may preferentially affect downstream inflammatory pathways that maintain the inflammatory response rather than the initial vascular events [31,32]. The distinct temporal profiles observed for S-8 and S-13 suggest that these extracts may influence different phases of the inflammatory response. S-8 predominantly affected the early vascular phase of serotonin-induced oedema, whereas S-13 exhibited a more sustained effect and was associated with stronger suppression of PGE_2_, TNF-α, and nitrotyrosine. This pattern may reflect differential modulation of early mediator-driven vascular permeability versus later prostaglandin-, cytokine-, and nitrosative stress-related pathways. In comparison, diclofenac and quercetin showed a different time-dependent profile, indicating that the investigated extracts may act through multimodal mechanisms rather than a single COX-dependent pathway.

The biochemical analyses further supported the anti-inflammatory properties of the investigated extracts. In the inflammatory control group, serum concentrations of TNF-α, PGE_2_, and nitrotyrosine increased markedly, confirming the development of acute systemic inflammation [33]. Treatment with S-13 resulted in the greatest reduction in PGE_2_ levels, exceeding the inhibitory effect observed for diclofenac. Since prostaglandin E_2_ is one of the principal mediators generated through the cyclooxygenase pathway, this observation is consistent with the hypothesis that S-13 may interfere with prostaglandin biosynthesis, thereby contributing to its prolonged anti-inflammatory activity [33]. Both S-8 and S-13 markedly decreased circulating TNF-α concentrations by approximately 75%, indicating a substantial modulation of the inflammatory cytokine response. Because TNF-α represents one of the key upstream mediators regulating inflammatory signalling, suppression of its production may contribute to the reduced tissue oedema observed throughout the experimental period [34]. Although the precise molecular targets remain to be elucidated, these findings suggest that the extracts influence inflammatory processes beyond simple inhibition of vascular permeability. Nitrotyrosine, a widely accepted biomarker of nitrosative stress [35], was also significantly reduced following treatment with S-8 and S-13. The observed decrease was comparable to that produced by diclofenac and substantially greater than that achieved with quercetin. These findings suggest that both extracts may attenuate oxidative and nitrosative stress associated with acute inflammation, thereby limiting cellular damage and supporting tissue recovery [36].

The differences in biological activity observed between S-8 and S-13 indicate that the anti-inflammatory effects of *S. officinale* extracts are likely determined by qualitative and quantitative differences in their phytochemical composition [5]. These observations are consistent with our previous phytochemical investigation, which demonstrated marked organ-specific variation in the distribution of bioactive constituents in *S. officinale*. It is therefore reasonable to assume that the superior pharmacological activity of S-8 and S-13 reflects a more favourable combination of anti-inflammatory phytochemicals acting through multiple complementary pathways.

The pronounced anti-inflammatory activity observed for extracts S-8 and S-13 is consistent with previous studies demonstrating the anti-inflammatory potential of *S. officinale*. Seigner et al. showed that a hydroalcoholic root extract suppresses the expression of major inflammatory mediators, including COX-2, VCAM-1, ICAM-1, and E-selectin, through inhibition of NF-κB signalling at multiple regulatory levels, providing the first mechanistic evidence for the anti-inflammatory action of *S. officinale* root extracts [37]. Our findings extend these observations by demonstrating that extracts prepared from different plant organs exhibit distinct anti-inflammatory profiles. In particular, the 70% ethanolic leaf extract (S-8) produced the strongest anti-exudative effect during the early vascular phase of serotonin-induced inflammation, whereas the 70% ethanolic flower extract (S-13) most effectively reduced serum PGE_2_, TNF-α, and nitrotyrosine concentrations, indicating a more pronounced influence on downstream inflammatory mediators.

The anti-inflammatory activity observed in the present study is further supported by previous investigations using different experimental models. Trifan et al. demonstrated that a hydroethanolic root extract of *S. officinale* and its major caffeic acid oligomers, including rosmarinic acid, significantly reduced the release of pro-inflammatory cytokines in LPS-stimulated human neutrophils, suggesting that phenolic constituents contribute substantially to the anti-inflammatory properties of comfrey roots [38]. Similarly, Wang et al. reported that a methanolic leaf extract of *S. officinale*, rich in rosmarinic acid, suppressed the expression of COX-2, iNOS, TNF-α, IL-1β, and IL-6 in LPS-stimulated RAW264.7 macrophages through inhibition of the NF-κB and MAPK signalling pathways [39]. In contrast to these studies, which investigated either root or leaf extracts using cell-based inflammatory models, the present work employed an in vivo serotonin-induced paw oedema model and directly compared extracts prepared from roots, leaves, and flowers. This approach demonstrated pronounced organ-specific differences in anti-inflammatory activity. Whereas the leaf extract S-8 exhibited the strongest anti-exudative effect during the early vascular phase of inflammation, the flower extract S-13 most effectively reduced serum PGE_2_, TNF-α, and nitrotyrosine concentrations, indicating differential modulation of inflammatory pathways by extracts derived from different plant organs.

Overall, the present findings indicate that *S. officinale* extracts exert a multimodal anti-inflammatory effect. Rather than acting through a single mechanism, the investigated extracts appear to combine early suppression of vascular inflammatory responses with inhibition of prostaglandin production, modulation of pro-inflammatory cytokines, and reduction in oxidative and nitrosative stress [33,40]. The molecular docking analysis presented in the subsequent section provides additional mechanistic support for these experimentally observed pharmacological effects.

The molecular docking analysis suggests that the antimicrobial and anti-inflammatory activities of the investigated *S. officinale* extracts are primarily associated with phenolic glycosides, particularly kaempferol-3-O-glucoside, hyperoside, and isoquercitrin [5]. Their high predicted binding affinities toward the selected molecular targets are likely attributable to the combination of a flavonoid scaffold and a glycosidic moiety, enabling the formation of multiple stabilising interactions within the enzyme active sites, including hydrogen bonds, hydrophobic contacts, π-interactions, and electrostatic interactions [41]. This observation is consistent with our previous molecular docking study on *Solidago canadensis*, in which flavonoids and caffeic acid derivatives likewise exhibited favourable interactions with inflammation-related molecular targets, supporting their contribution to the observed pharmacological activity [42].

Among the investigated phytochemicals, kaempferol-3-O-glucoside demonstrated the most favourable interaction profile with bacterial peptide deformylases. Of particular interest was its ability to establish a metal-acceptor interaction with the catalytic Zn^2+^ ion, which is essential for the enzymatic activity of this metalloprotease. In addition, the predicted docking pose indicated that the flavonoid moiety was accommodated within the hydrophobic region of the catalytic pocket, whereas the glucoside residue interacted with the surrounding polar amino acid residues. Such an orientation may contribute to stable ligand binding and suggests that kaempferol-3-O-glucoside could represent one of the principal contributors to the antimicrobial activity of the investigated extracts.

Isoquercitrin and hyperoside also exhibited favourable interaction profiles with peptide deformylase, supporting their possible contribution to the overall antimicrobial properties of *S. officinale*. Although rosmarinic acid displayed lower overall binding affinities than the flavonoid glycosides, it formed a particularly stable complex with the *Enterococcus faecalis* peptide deformylase (PDB ID: 2OS1), indicating that this phenolic acid may also contribute to the antibacterial activity of the extracts through complementary mechanisms.

Comparison with the co-crystallised inhibitor actinonin demonstrated that kaempferol-3-O-glucoside, isoquercitrin, and hyperoside share several common interaction features within the peptide deformylase active site. Although molecular docking alone cannot confirm enzyme inhibition, the observed similarities in binding mode support the hypothesis that these compounds may represent promising natural scaffolds for peptide deformylase inhibition.

Regarding the anti-inflammatory targets, hyperoside exhibited the strongest predicted binding affinity toward both cyclooxygenase-2 (COX-2) and 5-lipoxygenase (5-LOX) [43]. This favorable interaction profile is likely associated with the presence of multiple hydroxyl groups capable of forming an extensive hydrogen-bonding network, together with the aromatic flavonoid nucleus, which establishes additional π-mediated and hydrophobic interactions within the active sites of both enzymes.

Isoquercitrin and rosmarinic acid likewise demonstrated favourable predicted interactions with COX-2, whereas kaempferol-3-O-glucoside exhibited slightly lower binding affinity while maintaining a comparable interaction pattern. The glycosidic moiety of these flavonoids appears to enhance polar interactions with amino acid residues lining the binding pocket, whereas the flavonoid core contributes to complex stabilisation through hydrophobic and π-alkyl contacts. In contrast, the interaction profile of rosmarinic acid is likely dominated by its phenolic hydroxyl and carboxyl functional groups, which favour hydrogen-bonding and electrostatic interactions rather than extensive hydrophobic contacts.

Overall, the docking results are consistent with the experimental antimicrobial and anti-inflammatory findings and support the hypothesis that flavonoid glycosides may be responsible for the pharmacological activity of *S. officinale* extracts. Nevertheless, these computational predictions require further validation using enzymatic assays and additional mechanistic studies.

To the best of our knowledge, this is the first study providing a comparative evaluation of the antimicrobial and anti-inflammatory activities of thirteen extracts and fractions prepared from different organs of *S. officinale* in combination with molecular docking analysis. This integrated approach substantially extends the current understanding of the pharmacological potential of *S. officinale* and establishes a direct relationship between organ-specific phytochemical composition and multitarget biological activity.

Study Limitations and Future Perspectives. Although several *S. officinale* extracts demonstrated promising antimicrobial and anti-inflammatory activities, the present study has certain limitations. The biological assays were performed using complex plant extracts; therefore, the contribution of individual phytochemicals and their potential synergistic interactions cannot be established from the present data. In particular, although molecular docking identified several compounds with favourable predicted interactions with the selected targets, their contribution to the observed biological activities requires experimental confirmation using pure reference compounds at concentrations representative of those present in the investigated extracts. Furthermore, although molecular docking provided plausible mechanistic explanations for the observed pharmacological effects, these predictions require experimental validation by enzyme inhibition assays and additional in vitro and in vivo studies. Future investigations should also evaluate the toxicity and safety of the most active extracts and verify their efficacy in additional experimental models, including those of chronic inflammation and clinically relevant infections. Such studies will further clarify the therapeutic potential of *S. officinale* and support the development of standardised phytopharmaceutical preparations.

Despite the promising biological activities demonstrated in the present study, several safety-related aspects require further investigation. In particular, the presence of pyrrolizidine alkaloids (PAs) remains an important limitation for the therapeutic application of *S. officinale*. Future studies should therefore optimize extraction procedures to minimize PA content or selectively remove these compounds while preserving pharmacological activity. The safety implications of PAs also depend on the route of administration. While systemic exposure is associated with well-established hepatotoxic, genotoxic, and carcinogenic risks, available experimental evidence indicates relatively low percutaneous absorption of PAs through intact human skin [8,9]. According to our previous quantitative analysis of the same extract batch [5], the residual PA contents of the most active extracts S7, S8, and S13 were 26.32, 2.43, and 743.03 µg/g, respectively. Nevertheless, limited dermal absorption does not eliminate the need to control residual PA levels in topical preparations, and regulatory requirements emphasize restriction of PA exposure with respect to the amount applied and duration of treatment. Therefore, both residual PA content and pharmacological efficacy should be considered when selecting the most promising extracts for further topical development. Given these safety concerns, future pharmaceutical development should preferentially focus on PA-reduced or PA-depleted preparations enriched and standardized in phenolic constituents. In particular, phenolic compounds such as kaempferol-3-O-glucoside, hyperoside, isoquercitrin, and rosmarinic acid, which showed favourable predicted interactions with the investigated antimicrobial and anti-inflammatory targets, warrant further experimental evaluation as potential contributors to the biological activity of *S. officinale* extracts. Another limitation is the absence of cytotoxicity and cell viability assessment in mammalian cells. Although several extracts demonstrated pronounced antimicrobial and anti-inflammatory activities, these findings alone do not establish their cellular safety or therapeutic window. Therefore, the most active extracts, particularly S-7, S-8, and S-13, should be evaluated using relevant mammalian cell models to determine non-cytotoxic concentration ranges and establish whether their biological activities occur at concentrations compatible with cellular viability.

## 4. Materials and Methods

### 4.1. Plant Material and Preparation of Extracts

The *S. officinale* plant material and extracts used in the present study were identical to those described and phytochemically characterised in our previous publication [5]. Briefly, aerial parts (leaves and flowers) and roots were collected from wild populations in Tartu County, Estonia, botanically authenticated, air-dried at room temperature, and deposited at the Institute of Pharmacy, University of Tartu (voucher specimen No. Bor/Soff1).

Dried roots, leaves, and flowers were extracted with water, 40% ethanol, or 70% ethanol by infusion or maceration, depending on the extraction protocol [5]. For the hydroethanolic extracts, the dried plant materials were macerated with 40% or 70% aqueous ethanol at room temperature for 24 h. The extraction procedure was repeated twice using the same solvent volumes at each stage. Aqueous extracts were prepared by infusion at a plant material-to-water ratio of 1:10; the mixtures were heated to 100 °C for 15 min for leaves and flowers and 30 min for roots, followed by maceration for 24 h and filtration [5]. For the preparation of the polysaccharide and polyphenolic fractions, 50.0 g of dried *S. officinale* leaves and 100.0 g of dried roots were subjected to the same aqueous extraction procedure described above [5]. The resulting aqueous extracts were filtered and concentrated. Polysaccharides were precipitated from the concentrated extracts by adding three volumes of 96% ethanol and subsequently separated by filtration. The resulting polysaccharide precipitates were washed with ethanol and acetone and dried by lyophilization. The remaining supernatants were concentrated and dried to obtain the corresponding polyphenolic fractions. All extracts were concentrated under reduced pressure and freeze-dried to obtain dry extracts [5].

The extracts used throughout the present study were designated S1–S13. Samples S1–S5 were prepared from roots, S6–S10 from leaves, and S11–S13 from flowers. Water, 40% ethanol, and 70% ethanol crude extracts were obtained from all three plant organs, whereas polyphenolic and polysaccharide fractions were prepared only from aqueous root and leaf extracts (Table 6). The same batch of extracts characterised in the previous study was used in the present investigation to evaluate their antimicrobial and anti-inflammatory activities and to investigate the potential molecular mechanisms underlying these biological effects by molecular docking.

The extracts used in the present study were identical to those previously chemically characterized in our earlier work [5]. In that study, chromatographic–mass spectrometric analysis identified and quantified the major constituents of the root, leaf, and flower extracts, including kaempferol-3-O-glucoside, hyperoside, isoquercitrin, rosmarinic acid, caffeic acid, and ferulic acid, whereas intermedine, lycopsamine, and their N-oxides were identified as the major pyrrolizidine alkaloids. The quantitative phytochemical profiles revealed marked differences in the distribution and abundance of phenolic constituents among the root-, leaf-, and flower-derived preparations, depending on the plant organ, extraction solvent, and fractionation procedure. These previously established chemical profiles guided the selection of compounds for molecular docking and were used to support the interpretation of the organ- and extract-specific biological activities observed in the present study. Detailed quantitative data for the individual extracts are provided in Ref. [5].

### 4.2. Antimicrobial Activity

Antimicrobial activity was evaluated in the Educational and Scientific Laboratory of the Department of Infectious Animal Diseases, Dnipro State Agrarian and Economic University (Dnipro, Ukraine). A collection of reference strains was used in accordance with the experimental design. The tested microorganisms were identified based on their morphological, cultural, and biochemical characteristics. The bacterial panel included *Salmonella enterica* subsp. *enterica* serovar Typhimurium (UNCSM-014), *Klebsiella pneumoniae* (ATCC 13883), *Escherichia coli* (ATCC 8739), *Enterococcus faecalis* (ATCC 29212), *Staphylococcus aureus* (ATCC 25923), and *Listeria monocytogenes* (ATCC 19112) (Table 1). Bacterial cultures were maintained and cultivated using standard nutrient media, including meat-peptone broth (MPB) and meat-peptone agar (MPA), under appropriate incubation conditions [43].

*Broth serial dilution assay.* The antimicrobial activity of the extracts was evaluated using a broth serial dilution method. The extracts were initially dissolved in dimethyl sulfoxide (DMSO) to obtain stock solutions and were subsequently diluted with the culture medium to the required test concentrations. Serial two-fold dilutions were prepared to obtain final extract concentrations of 16, 8, 4, and 2 µg/mL. These concentrations corresponded to the first (16 µg/mL), second (8 µg/mL), third (4 µg/mL), and fourth (2 µg/mL) serial dilutions, respectively. The final concentration of DMSO in the assay was 1% (*v*/*v*). A standardized bacterial suspension corresponding to 0.5 McFarland was used as the microbial inoculum. All in vitro antimicrobial assays were performed in independent triplicates to ensure reproducibility.

Ampicillin trihydrate at a concentration of 2 µg/mL was used as the reference positive antimicrobial control. A vehicle control containing 1% (*v*/*v*) DMSO without the extract served as the negative control. In addition, inoculated nutrient medium without the extract or reference antimicrobial agent was included as a growth control, whereas uninoculated nutrient medium was used as a sterility control. All experimental and control samples were incubated at 37 °C for 18–24 h.

*Assessment of Antimicrobial Activity and CFU-Based Inhibitory Endpoint.* Following incubation, bacterial growth was evaluated at each tested concentration. Quantitative bacterial growth was expressed as colony-forming units (CFU/mL) and used for comparative assessment of the inhibitory effects of the investigated extracts. For Table 1, bacterial growth at the fourth serial dilution (2 µg/mL) was expressed as CFU × 10^9^/mL to enable direct comparison among the investigated samples and bacterial strains. For comparative visualization of the concentration-dependent antimicrobial effects, an operational CFU-based inhibitory endpoint was applied. A viable bacterial count of ≤1.0 × 10^9^ CFU/mL was predefined as the threshold indicating pronounced growth inhibition. The lowest tested extract concentration (16, 8, 4, or 2 µg/mL) reaching this threshold was designated as the CFU-based inhibitory concentration. Samples that did not reach the predefined endpoint at 16 µg/mL were designated as >16 µg/mL, whereas samples reaching the endpoint at the lowest tested concentration were designated as ≤2 µg/mL. These values were used exclusively for comparative visualization and ranking and should not be interpreted as conventional MIC values.

*Heatmap construction.* Because the heatmap was intended for comparative ranking rather than formal MIC determination, an operational inhibitory endpoint was applied to the quantitative CFU data. For this analysis, CFU ≤ 1.0 × 10^9^/mL was predefined as the threshold indicating an inhibitory effect. The lowest tested concentration (16, 8, 4, or 2 µg/mL) meeting this criterion was assigned as the corresponding MIC-like inhibitory concentration. Samples that did not reach the predefined inhibitory endpoint at 16 µg/mL were designated as >16 µg/mL, whereas samples meeting the criterion at the lowest tested concentration were designated as ≤2 µg/mL. These MIC-like values were used exclusively for comparative visualization and ranking and should not be interpreted as formal MIC values determined by complete absence of visible microbial growth. To provide an integrated measure of antimicrobial activity across the bacterial panel, an Overall Activity Score was calculated as the geometric mean of the MIC-like inhibitory concentrations obtained for each extract. For ranking purposes, values of >16 µg/mL were assigned a numerical value of 32 µg/mL, whereas values of ≤2 µg/mL were assigned a value of 2 µg/mL. These substitutions were used solely for calculation of the ranking score. Lower Overall Activity Score values indicated greater overall antimicrobial activity [44,45].

The heatmap was generated using Python (version 3.13.5) and the Matplotlib library (version 3.10.8). No normalization or standardization procedures, including z-score transformation, were applied. The heatmap was used exclusively for comparative visualization; therefore, hierarchical clustering or other clustering algorithms were not performed, and the extracts and bacterial strains were displayed in a predefined order.

### 4.3. Anti-Inflammatory Activity

The anti-inflammatory activity of the *S. officinale* extracts was evaluated using the serotonin-induced paw oedema model in rats, in accordance with the recommendations for the preclinical evaluation of non-steroidal anti-inflammatory drugs (NSAIDs). Acute inflammation was induced by subplantar injection of 0.1 mL of a 0.5% serotonin solution (Thermo Fisher Scientific, Waltham, MA, USA) into the hind paw. The anti-inflammatory effect was evaluated by monitoring the development of serotonin-induced paw oedema over a 5 h observation period, with measurements performed at 1, 3, and 5 h after serotonin administration [46].

The experiments were performed on adult outbred white rats (160–180 g) maintained under standard laboratory conditions with free access to food and water. All experimental procedures complied with the European Convention for the Protection of Vertebrate Animals Used for Experimental and Other Scientific Purposes (Strasbourg, 1986) [47] and the Law of Ukraine “On Protection of Animals from Cruelty” (No. 3447-IV, 21 February 2006) [48,49]. The experimental protocol was approved by the Bioethics Commission of the ZSMPU (protocol 4 dated 12 March 2026).

The animals were randomly assigned to 17 experimental groups (*n* = 8 per group). Thirteen groups received the tested *S. officinale* extracts, one group served as the inflammatory control (vehicle plus serotonin), one group received diclofenac sodium (8 mg/kg, Diclofenac-Darnitsa, Kyiv, Ukraine) as the reference anti-inflammatory drug, one group received quercetin (25 mg/kg, Borshchagov Chemical and Pharmaceutical Plant, Kyiv, Ukraine) as a reference phytochemical, and one control group received water for injection without serotonin administration. The tested extracts were administered intragastrically as aqueous solutions at a dose of 100 mg/kg body weight. This dose was used as a fixed comparative screening dose to enable direct comparison of the investigated extracts in a single in vivo model and was selected as a conservative oral dose within the range reported for *S. officinale* preparations in preclinical studies. Published in vivo studies have reported oral administration of *S. officinale* preparations at 100–400 mg/kg in a mouse colitis model and 500 mg/kg in a rat paw oedema model, supporting the use of 100 mg/kg as a lower comparative screening dose [4,12]. Diclofenac sodium (8 mg/kg) and quercetin (25 mg/kg) were administered intragastrically under the same conditions. All treatments were given 1 h before serotonin injection. Animals in the control group received an equivalent volume of water for injection.

The initial paw volume was measured immediately before serotonin administration using a plethysmometer. Following serotonin injection, paw volume was measured at 1, 3, and 5 h to evaluate the time course of serotonin-induced oedema and the anti-exudative effects of the investigated treatments. At each observation time, the increase in paw volume (ΔV, mL) was calculated as the difference between the paw volume measured at the corresponding time point and the initial paw volume [43,46]. Anti-exudative activity (AA, %) was calculated relative to the inflammatory control group at each corresponding observation time using the following equation:

AA (%) = [(ΔVcontrol − ΔVtreated)/ΔVcontrol] × 100,(1)

where ΔVcontrol is the mean increase in paw volume in the inflammatory control group and ΔVtreated is the mean increase in paw volume in the corresponding treatment group at the same observation time. Higher AA values indicate greater inhibition of serotonin-induced paw oedema.

Following completion of the experiment, blood samples were collected for biochemical analysis. To prevent analytical bias, all serum assays were performed by an investigator blinded to the experimental group assignments. Serum levels of prostaglandin E_2_ (PGE_2_), nitrotyrosine, and tumour necrosis factor-α (TNF-α) were determined using commercial ELISA kits (Elabscience^®^, Houston, TX, USA) validated for rat serum, according to the manufacturer’s instructions. Specifically, a species-specific kit was used for Rat TNF-α, whereas universal kits were used for the species-independent small molecules PGE_2_ and nitrotyrosine. Measurements were performed using an ImmunoChem-2100 microplate reader (HTI, North Attleboro, MA, USA).

### 4.4. Molecular Docking

Molecular docking studies were performed to investigate potential interactions between the major phytochemical constituents of *Symphytum officinale* extracts and protein targets implicated in inflammatory processes and bacterial viability. The three-dimensional crystal structures of the target proteins were retrieved from the Protein Data Bank (PDB, https://www.rcsb.org). Proteins involved in inflammation included cyclooxygenase-2 (COX-2; PDB ID: 5IKR) and 5-lipoxygenase (5-LOX; PDB ID: 3O8Y). To evaluate the potential antimicrobial activity, peptide deformylases from *Escherichia coli* (PDB ID: 1LRU), *Staphylococcus aureus* (PDB ID: 1Q1Y), and *Enterococcus faecalis* (PDB ID: 2OS1) were selected as bacterial targets. The three-dimensional structures of the investigated ligands were generated using MarvinSketch version 6.3.0 (ChemAxon, Budapest, Hungary), HyperChem version 8 (Hypercube Inc., Gainesville, FL, USA), and AutoDockTools version 1.5.6. Protein structures were prepared for docking by removing crystallographic water molecules, adding hydrogen atoms, and assigning Kollman charges using Discovery Studio version 4.0 (Dassault Systèmes BIOVIA, San Diego, CA, USA) and AutoDockTools version 1.5.6. Docking simulations were carried out using AutoDock Vina, which predicts ligand–protein binding by evaluating the energetic and spatial complementarity between ligands and receptor binding sites. The predicted binding affinity (kcal/mol) was used to compare ligand interactions with the selected molecular targets.

### 4.5. Statistical Analysis

Statistical analysis of the results was performed using BioStat LE software version 7.3 (AnalystSoft Inc., Walnut, USA, 2019) and MedCalc version 16.4 (MedCalc Software Ltd., Ostend, Belgium, 2016) [44,45]. The data were evaluated using Student’s t-test and one-way analysis of variance (ANOVA). Differences were considered statistically significant at *p* < 0.05 [45]. Quantitative continuous data are presented as mean ± standard deviation (SD).

## 5. Conclusions

The present study demonstrated that *S. officinale* extracts exhibit both antimicrobial and anti-inflammatory activities, although their pharmacological effects varied with the plant organ used and the extraction method. Among the investigated samples, extracts S-7 and S-13 exhibited the broadest antimicrobial activity against multidrug-resistant bacterial strains, while S-2 showed pronounced selective inhibitory effects. These findings indicate that S-7 and S-13 may be considered the most promising natural antimicrobial candidates for further phytochemical and pharmacological investigation. In the anti-inflammatory study, extract S-8 showed the highest anti-exudative activity during the early phase of serotonin-induced inflammation, whereas extract S-13 most effectively suppressed prostaglandin E_2_ production and markedly reduced TNF-α and nitrotyrosine levels, demonstrating pronounced modulation of inflammatory mediators. Molecular docking analysis supported the experimental findings by suggesting that flavonoid glycosides, particularly kaempferol-3-O-glucoside, may contribute to the antimicrobial activity of the studied extracts through interactions with bacterial peptide deformylases. Likewise, the predicted interactions of hyperoside, isoquercitrin, and rosmarinic acid with cyclooxygenase-2 (COX-2) and 5-lipoxygenase (5-LOX) provide a plausible molecular basis for the observed anti-inflammatory effects. Overall, the obtained results indicate that *S. officinale* represents a promising source of biologically active phytochemicals with multitarget antimicrobial and anti-inflammatory properties, warranting further pharmacological and mechanistic investigations of the studied extracts. However, further pharmaceutical development of these extracts requires careful control of pyrrolizidine alkaloid content, and PA-reduced or PA-depleted preparations should be prioritized to minimize potential safety risks.

## Figures and Tables

**Table 1:** Summary results of the antimicrobial activity of extracts and polysaccharide fractions of *S. officinale* in vitro.

Extract/ Fraction	Bacterial Growth at the Fourth Dilution (CFU × 10^9^/mL)					
	*S. typhimurium* UNSCM-014	*S. aureus* ATCC 25923	*E. faecalis* ATCC 29212	*E. coli* ATCC 8739	*K. pneumoniae* ATCC 13883	*L. monocytogenes* ATCC 19112
Negative control	31.13	38.22	21.32	18.31	34.81	36.67
Positive control	5.24	6.27	1.74	5.34	–	4.16
S-1	2.71	3.55	1.60	3.13	1.55	1.85
S-2	1.95	1.16	0.75	1.43	2.53	0.63
S-3	2.29	1.73	1.72	2.25	2.55	1.43
S-4	3.39	2.73	1.94	2.77	2.16	2.45
S-5	2.15	0.72	0.85	1.59	1.88	0.62
S-6	3.27	1.81	1.54	2.88	2.16	0.68
S-7	2.32	0.73	0.86	0.32	2.13	0.54
S-8	1.98	1.17	1.21	1.95	1.72	1.43
S-9	2.95	1.19	2.52	2.73	2.19	1.74
S-10	1.61	1.84	1.17	1.82	2.45	0.72
S-11	3.64	1.83	2.66	2.65	3.15	3.13
S-12	3.32	1.57	1.94	2.24	2.78	3.57
S-13	1.62	0.69	0.74	0.28	1.83	0.37

Note. Values represent viable bacterial counts (CFU × 10^9^/mL) determined at the fourth serial dilution of the tested samples, corresponding to an extract concentration of 2 µg/mL. Lower CFU values indicate greater antimicrobial activity. Samples S-1–S-5 were obtained from roots, S-6–S-10 from leaves, and S-11–S-13 from flowers; detailed extraction and fractionation procedures are provided in the Section 4.1. Plant Material and Preparation of Extracts. Ampicillin trihydrate (2 µg/mL) and 1% DMSO were used as the positive and vehicle controls, respectively. Complete concentration-dependent CFU profiles at 2, 4, 8, and 16 µg/mL are provided in Figure S1.

**Table 2:** Changes in rat paw volume following serotonin-induced inflammation and treatment with *Symphytum officinale* extracts (*n* = 8 per group).

Group	Initial Paw Volume, mL	Increase in Paw Volume, mL					
		ΔV After 1 h	AA, %	ΔV After 3 h	AA, %	ΔV After 5 h	AA, %
S-1	1.12 ± 0.20	0.76 ± 0.09	13.30	1.10 ± 0.09 *	30.62	1.80 ± 0.10	6.84
S-2	1.15 ± 0.21	0.82 ± 0.07	7.06	1.13 ± 0.11 *	27.37	1.54 ± 0.15 *	20.56
S-3	1.24 ± 0.24	0.77 ± 0.07	12.73	1.14 ± 0.14	26.17	1.65 ± 0.14	14.89
S-4	1.18 ± 0.24	0.69 ± 0.04 *	21.91	1.11 ± 0.09 *	29.47	1.81 ± 0.26	6.28
S-5	1.20 ± 0.23	0.72 ± 0.08	17.57	1.31 ± 0.22	9.59	1.81 ± 0.26	6.48
S-6	1.17 ± 0.22	0.53 ± 0.07 *	39.27	0.91 ± 0.13 *	48.79	1.39 ± 0.26 *	28.03
S-7	1.25 ± 0.20	0.53 ± 0.07 *	40.21	0.95 ± 0.12 *	45.17	1.42 ± 0.10 *	26.80
S-8	1.13 ± 0.27	0.46 ± 0.07 *	47.50	1.11 ± 0.10	29.46	1.58 ± 0.14	18.61
S-9	1.22 ± 0.20	0.61 ± 0.08 *	30.26	0.95 ± 0.15 *	45.00	1.64 ± 0.19	15.32
S-10	1.20 ± 0.20	0.64 ± 0.08 *	27.22	0.90 ± 0.15 *	49.63	1.54 ± 0.18	20.32
S-11	1.18 ± 0.22	0.49 ± 0.05 *	43.67	1.06 ± 0.07 *	34.59	1.67 ± 0.15	13.90
S-12	1.14 ± 0.17	0.56 ± 0.10 *	35.83	1.04 ± 0.30	35.66	1.58 ± 0.22	18.62
S-13	1.17 ± 0.14	0.63 ± 0.19	27.82	0.89 ± 0.09 *	50.85	1.21 ± 0.11 *	37.32
Diclofenac sodium	1.39 ± 0.28	0.61 ± 0.18 *	30.96	0.86 ± 0.09 *	39.60	1.34 ± 0.13 *	30.66
Quercetin	1.18 ± 0.18	0.74 ± 0.21	15.58	1.04 ± 0.10 *	26.14	1.51 ± 0.16 *	21.85
Inflammatory control	1.23 ± 0.13	0.88 ± 0.17	-	1.41 ± 0.25	-	1.94 ± 0.31	
Control group	1.25 ± 0.17	-	-	-	-	-	

Note: Data are expressed as mean ± standard deviation (SD), *n* = 8 animals per group. ΔV represents the increase in paw volume relative to the initial paw volume measured before serotonin administration. AA (%) represents anti-exudative activity, calculated relative to the inflammatory control group; higher AA values indicate greater inhibition of serotonin-induced paw oedema. The inflammatory control group received vehicle followed by serotonin, whereas the control group received vehicle without serotonin-induced inflammation. Diclofenac sodium and quercetin were used as reference treatments. * indicates a statistically significant difference compared with the inflammatory control group (*p* ≤ 0.05).

**Table 3:** Serum levels of inflammatory biomarkers (TNF-α, PGE_2_, and nitrotyrosine) in rats with serotonin-induced paw oedema (*n* = 8 per group).

Group	TNF Alpha, pg/mL	Inhibition, %	PGE2, pg/mL	Inhibition, %	Nitrotyrosine, ng/mL	Inhibition, %
S-1	459.40 ± 2.45 *	14.41%	1225.78 ± 15.33 *	20.44%	53.41 ± 4.07	14.97%
S-2	507.37 ± 3.65	5.47%	1310.21 ± 24.09	14.96%	57.77 ± 4.26	8.02%
S-3	467.96 ± 4.95	12.81%	1421.60 ± 24.02	7.73%	49.77 ± 4.07 *	20.76%
S-4	508.89 ± 6.83	5.18%	1134.94 ± 41.09 *	26.33%	46.47 ± 4.12 *	26.02%
S-5	425.00 ± 7.14 *	20.81%	1323.46 ± 23.90	14.10%	47.48 ± 4.25 *	24.41%
S-6	253.45 ± 1.81 *	52.78%	739.17 ± 25.06 *	52.02%	32.22 ± 2.16 *	48.70%
S-7	262.02 ± 6.21 *	51.18%	740.48 ± 42.26 *	51.94%	32.03 ± 3.35 *	49.01%
S-8	131.46 ± 2.55 *	75.51%	358.09 ± 9.43 *	76.76%	16.07 ± 1.65 *	74.41%
S-9	387.20 ± 9.55 *	27.86%	1334.54 ± 30.78	13.38%	44.61 ± 4.23 *	28.98%
S-10	258.99 ± 2.46 *	51.74%	741.34 ± 35.94 *	51.88%	30.71 ± 2.02 *	51.10%
S-11	418.70 ± 9.53	21.99%	1162.09 ± 28.07	24.57%	44.55 ± 4.35	29.07%
S-12	404.50 ± 5.98 *	24.63%	1288.12 ± 18.58	16.39%	45.52 ± 3.76	27.53%
S-13	131.97 ± 2.35 *	75.41%	357.82 ± 18.70 *	76.77%	16.43 ± 2.17 *	73.84%
Diclofenac sodium	129.81 ± 2.29 *	75.81%	374.83 ± 8.88 *	75.67%	15.98 ± 0.82 *	74.56%
Quercetin	260.59 ± 3.72 *	51.45%	741.65 ± 21.70	51.86%	26.29 ± 6.93 *	58.14%
Inflammatory control	536.71 ± 11.60	-	1540.65 ± 55.90	-	62.81 ± 7.81	-
Control group	39.93 ± 2.38	-	315.61 ± 8.00	-	11.43 ± 0.51	-

Note: Data are expressed as mean ± standard deviation (SD), *n* = 8 animals per group. Inhibition (%) represents the percentage reduction in the corresponding inflammatory biomarker relative to the inflammatory control group. TNF-α, tumour necrosis factor-α; PGE_2_, prostaglandin E_2_. The inflammatory control group received vehicle followed by serotonin, whereas the control group received vehicle without serotonin-induced inflammation. Diclofenac sodium and quercetin were used as reference treatments. * indicates a statistically significant difference compared with the inflammatory control group (*p* ≤ 0.05).

**Table 4:** Predicted binding energies (kcal/mol) of major *Symphytum officinale* phytochemicals toward antimicrobial and anti-inflammatory protein targets.

Compound	Antimicrobial Targets			Anti-Inflammatory Targets	
	1LRU	1Q1Y	2OS1	3O8Y	5IKR
Kaempferol-3-O-glucoside	−5.6	−10.9	−9.8	−8.6	−8.2
Hyperoside	−5.4	−9.2	−9.1	−9.2	−10.3
Isoquercitrin	−9.5	−10.1	−9.7	−8.4	−9.5
Rosmarinic acid	−4.1	−8.1	−9.1	−7.5	−9.5
Intermedine	−3.9	−7.5	−7.4	−6.5	−7.5
Lycopsamine	−3.9	−7.5	−7.4	−6.5	−7.5
Intermedine N-oxide	−4.1	−7.4	−7.2	−6.8	−6.7
Lycopsamine N-oxide	−3.8	−6.3	−6.8	−6.9	−6.8
Caffeic acid	−3.2	−6.4	−6.7	−6.5	−7.5
Ferulic acid	−3.5	−5.8	−6.7	−6.6	−7.6

Notes: Binding energies are expressed in kcal/mol; more negative values indicate stronger predicted ligand–target binding affinity. PDB IDs 1LRU, 1Q1Y, and 2OS1 correspond to bacterial peptide deformylases from *E. coli*, *S. aureus*, and *E. faecalis*, respectively; 3O8Y corresponds to 5-LOX and 5IKR to COX-2.

**Table 5:** Heatmap of MIC-like inhibitory concentrations of *S. officinale* extracts and polysaccharide fractions against antibiotic-resistant microorganisms (*in vitro*).

Group	Microorganisms *						Overall Activity Score **	MIC (µg/mL)	
	*S. enterica* (*typhimurium*) UNSCM-014	*S. aureus* ATCC 25923	*E. faecalis* ATCC 29212	*E. coli* ATCC 8739	*K. pneumoniae* ATCC 13883	*L. monocytogenes* ATCC 19112			
**S-1**	>16	8	>16	>16	>16	>16	>16		≤2
**S-2**	>16	4	≤2	>16	>16	≤2	9.0		4
**S-3**	>16	>16	>16	>16	>16	>16	>16		8
**S-4**	>16	>16	>16	>16	>16	>16	>16		16
**S-8**	>16	>16	>16	>16	>16	16	>16		>16
**S-5**	>16	≤2	≤2	>16	>16	≤2	8.0		
**S-6**	>16	16	>16	>16	>16	≤2	>16	Lower value = Higher activity (more potent)	
**S-7**	>16	≤2	≤2	≤2	>16	≤2	5.0		
**S-9**	>16	8	4	>16	>16	>16	>16		
**S-10**	>16	4	8	>16	>16	≤2	11.3	Higher value = Lower activity (less potent)	
**S-11**	>16	>16	8	>16	>16	8	>16		
**S-12**	>16	>16	>16	>16	>16	>16	>16		
**S-13**	>16	≤2	≤2	≤2	>16	≤2	5.0		

Note: * Lower values indicate higher antimicrobial activity. MIC-like values were assigned from serial dilution data (16, 8, 4, and 2 µg/mL) using CFU ≤ 1.0 × 10^9^ as the operational endpoint; ≤2 µg/mL indicates inhibition at the lowest tested concentration. ** Overall Activity Score = geometric mean of MIC-like values (lower score = higher overall activity).

**Table 6:** Description and preparation of the *Symphytum officinale* extracts and fractions used in the present study [5].

Sample	Plant Organ	Extraction Solvent	Extraction/ Fractionation Method	Preparation Method	Yield of the Extract, % [5]
S-1	Roots	Water	Crude extract	Infusion	20.38 *
S-2	Roots	40% ethanol	Crude extract	Maceration	18.21
S-3	Roots	70% ethanol	Crude extract	Maceration	14.42
S-4	Roots	Water	Polyphenolic fraction	Ethanol precipitation and fractionation	8.62
S-5	Roots	Water	Polysaccharide fraction	Ethanol precipitation	9.87
S-6	Leaves	Water	Crude extract	Infusion	23.12 *
S-7	Leaves	40% ethanol	Crude extract	Maceration	20.43
S-8	Leaves	70% ethanol	Crude extract	Maceration	16.93
S-9	Leaves	Water	Polyphenolic fraction	Ethanol precipitation and fractionation	13.54
S-10	Leaves	Water	Polysaccharide fraction	Ethanol precipitation	7.51
S-11	Flowers	Water	Crude extract	Infusion	24.39
S-12	Flowers	40% ethanol	Crude extract	Maceration	21.38
S-13	Flowers	70% ethanol	Crude extract	Maceration	17.31

Note: Extract and fraction yields (%) were calculated on a dry-weight basis relative to the initial mass of the corresponding dry plant material: Yield (%) = (mass of the obtained dry extract or fraction / initial mass of dry plant material) × 100. * Due to the presence of mucilage, separation from the plant material was difficult.

## Data Availability

The original contributions presented in this study are included in the article/Supplementary Material. Further inquiries can be directed to the corresponding author(s).

## Supplementary Materials

The following supporting information can be downloaded at https://www.mdpi.com/article/10.3390/ijms27188060/s1.
