# Supplementary material for: Anti-Inflammatory and Antimicrobial Activities of Extracts Developed from Symphytum officinale Leaves, Flowers, and Roots: Experimental Evaluation and Molecular Docking Study
Source: Int J Mol Sci. 2026 Sep 10;27(18):8060. doi: 10.3390/ijms27188060 (PMC13606896; doi:10.3390/ijms27188060)
Supplement: Supplementary file 1 [file ijms-27-08060-s001.zip › ijms-4484300-supplementary.pdf]

Supplementary information

Anti-Inflammatory and Antimicrobial Activities of Extracts Developed from Symphytum Officinale Leaves, Flowers, and Roots: Experimental Evaluation and Molecular Docking Study

Oleh Koshovyi [1, 2*], Getter Dolgošev [1], Andrii Kaplaushenko [2], Oleksandr Panasenko [2], Volodymyr Zazharskyi [3], Yuriy Karpenko [2], Roman Shcherbyna [2], Andriy Hotsulia [2], Mariia Shanaida [4], Ivo Laidmäe [1], Jyrki Heinämäki [1] and Ain Raal [1]

[1] **Institute of Pharmacy, Faculty of Medicine, University of Tartu, 50411 Tartu, Estonia; oleh.koshovyi@ut.ee (O.K.), getter.dolgosev@ut.ee (G.D.), ivo.laidmae@ut.ee (I.L.), jyrki.heinamaki@ut.ee (J.H.) and ain.raal@ut.ee (A.R.)**

[2] Zaporizhzhia State Medical and Pharmaceutical University, 69035 Zaporizhzhia, Ukraine; kaplaushenko.a.g@zsmu.edu.ua (A.K.); panasenko.o.i@zsmu.edu.ua (O.P.); karpenko.y.v@zsmu.edu.ua (Yu.K.), shcherbyna.r.o@zsmu.edu.ua (R.Sh.), hotsulia.a.s@zsmu.edu.ua (H.A.)

[3] Dnipro State Agrarian and Economic University, 49009 Dnipro, Ukraine; zazharskyi.v.v@dsau.dp.ua (V.Z.)

[4] I. Horbachevsky Ternopil National Medical University, 46001 Ternopil, Ukraine; shanayda@tdmu.edu.ua (M.Sh.)

* Correspondence: oleh.koshovyi@ut.ee (O.K.); Tel.: +372 5027574 (Nooruse 1, Tartu 50411, Estonia)

Figure S1. Concentration-dependent CFU profiles of *S. officinale* extracts and polysaccharide fractions against the tested bacterial strains: (A) *Salmonella enterica* serovar Typhimurium UNSCM-014; (B) *Staphylococcus aureus* ATCC 25923; (C) *Enterococcus faecalis* ATCC 29212; (D) *Escherichia coli* ATCC 8739; (E) *Klebsiella pneumoniae* ATCC 13883; and (F) *Listeria monocytogenes* ATCC 19112. CFU values were determined after incubation with the tested samples at concentrations of 2, 4, 8, and 16 μg/mL.

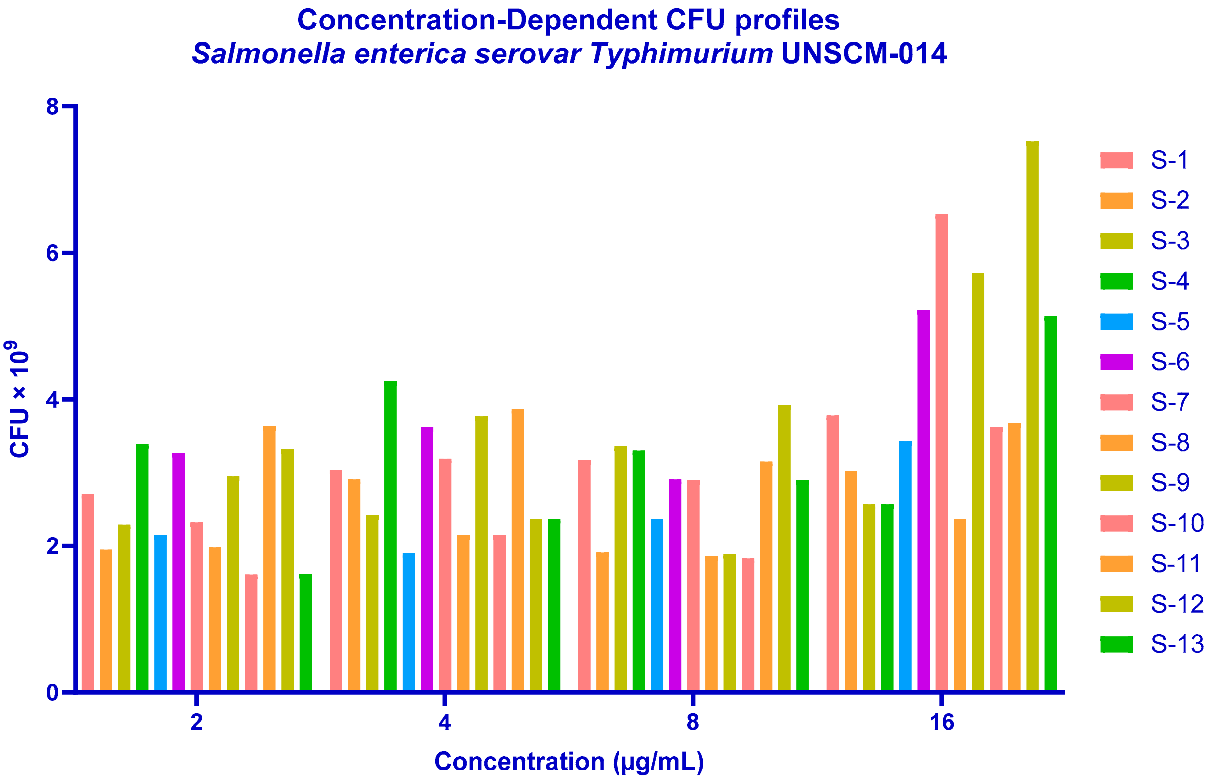

Figure S1.A

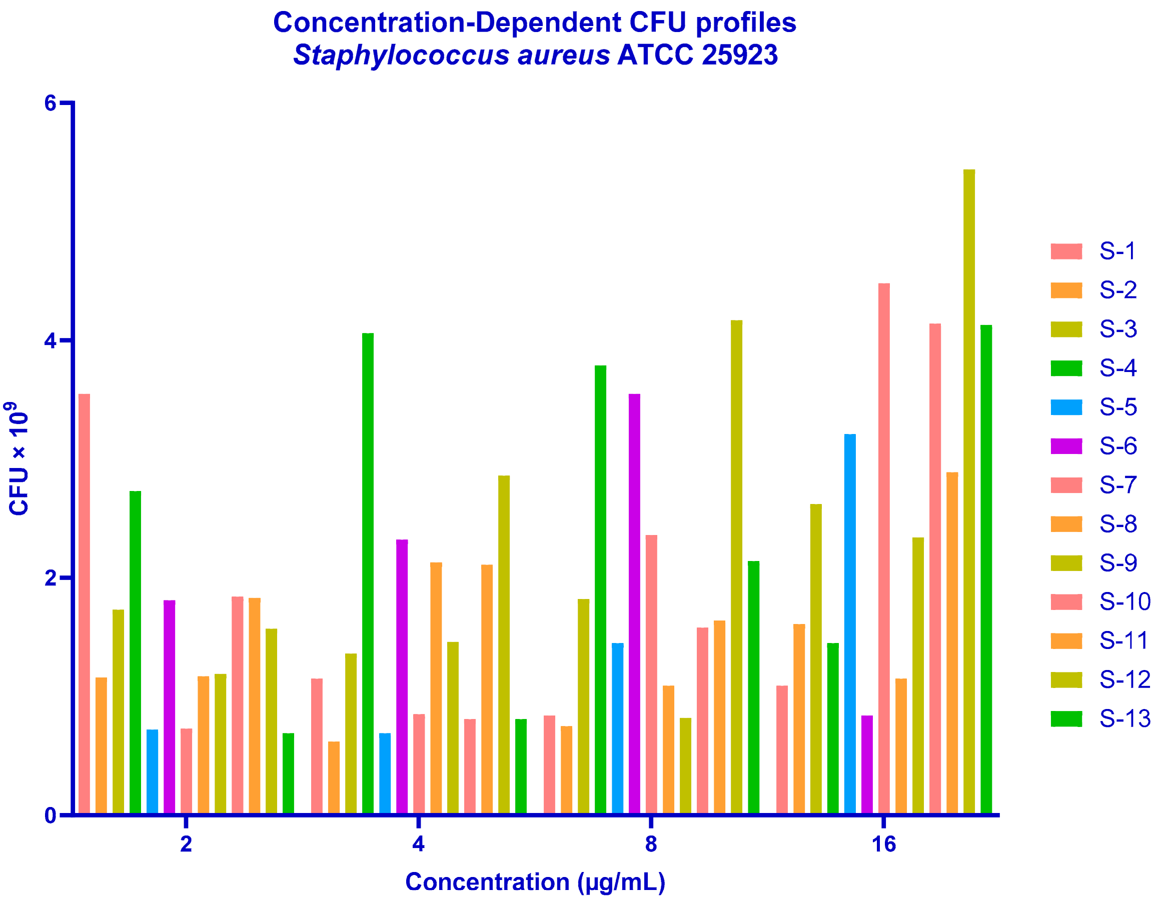

Figure S1.B

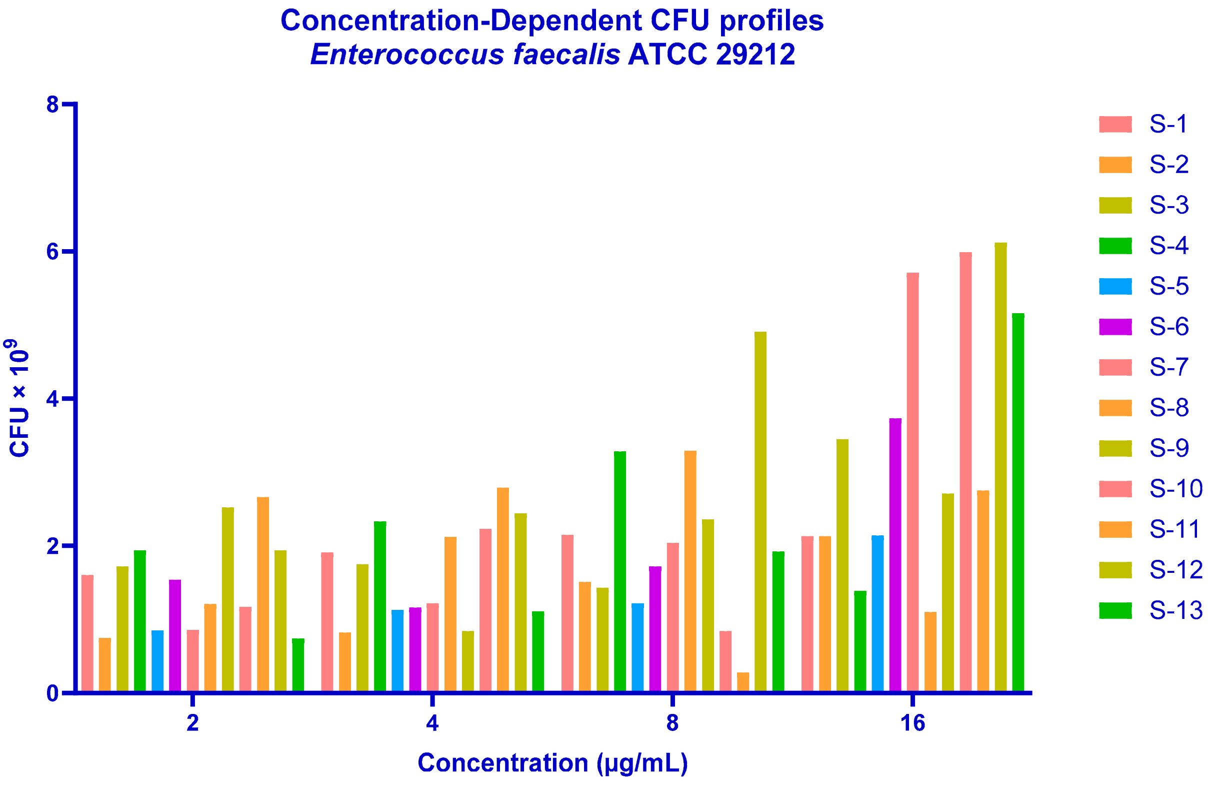

Figure S1.C

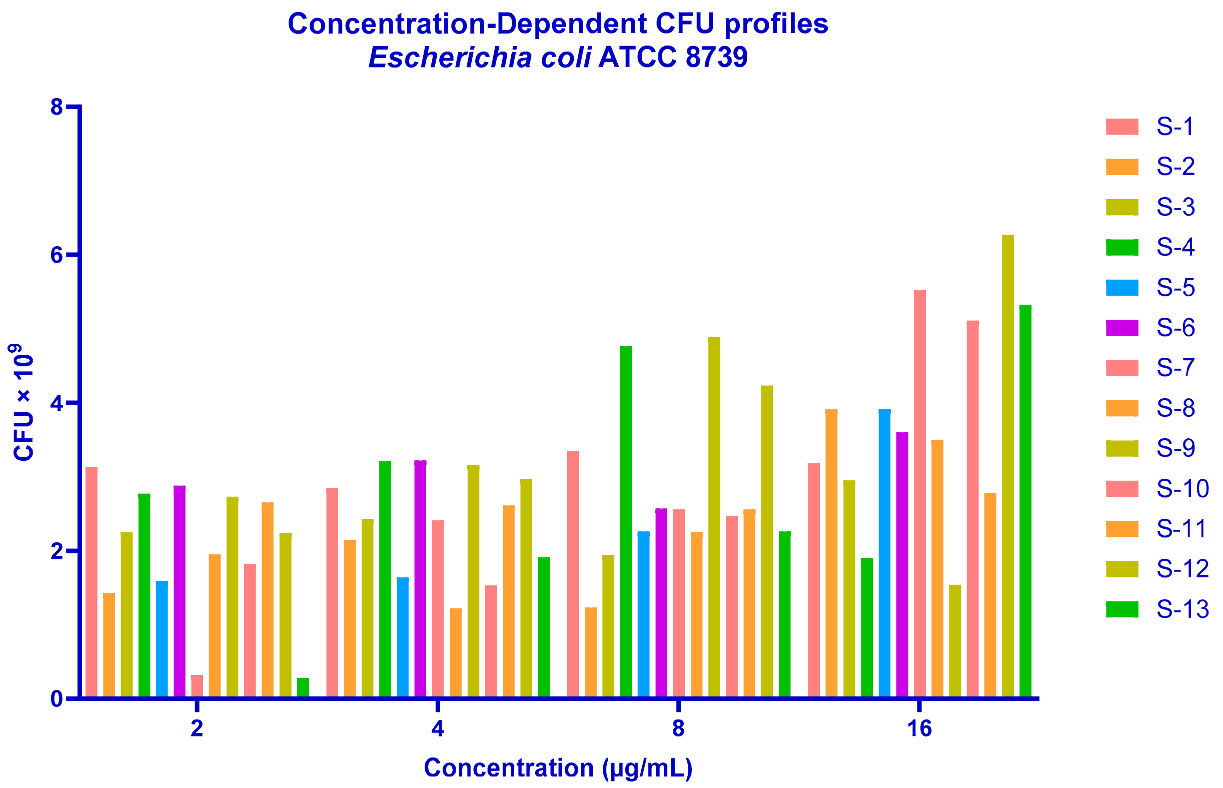

Figure S1.D

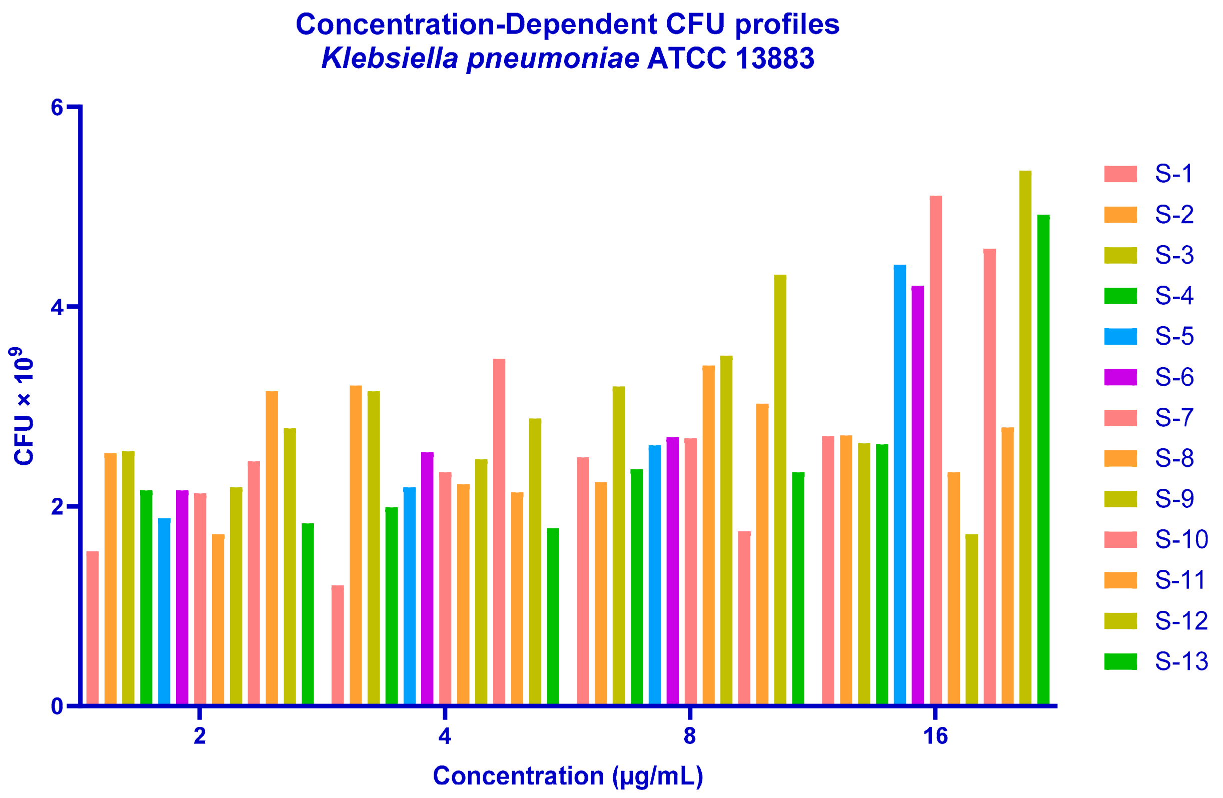

Figure S1.E

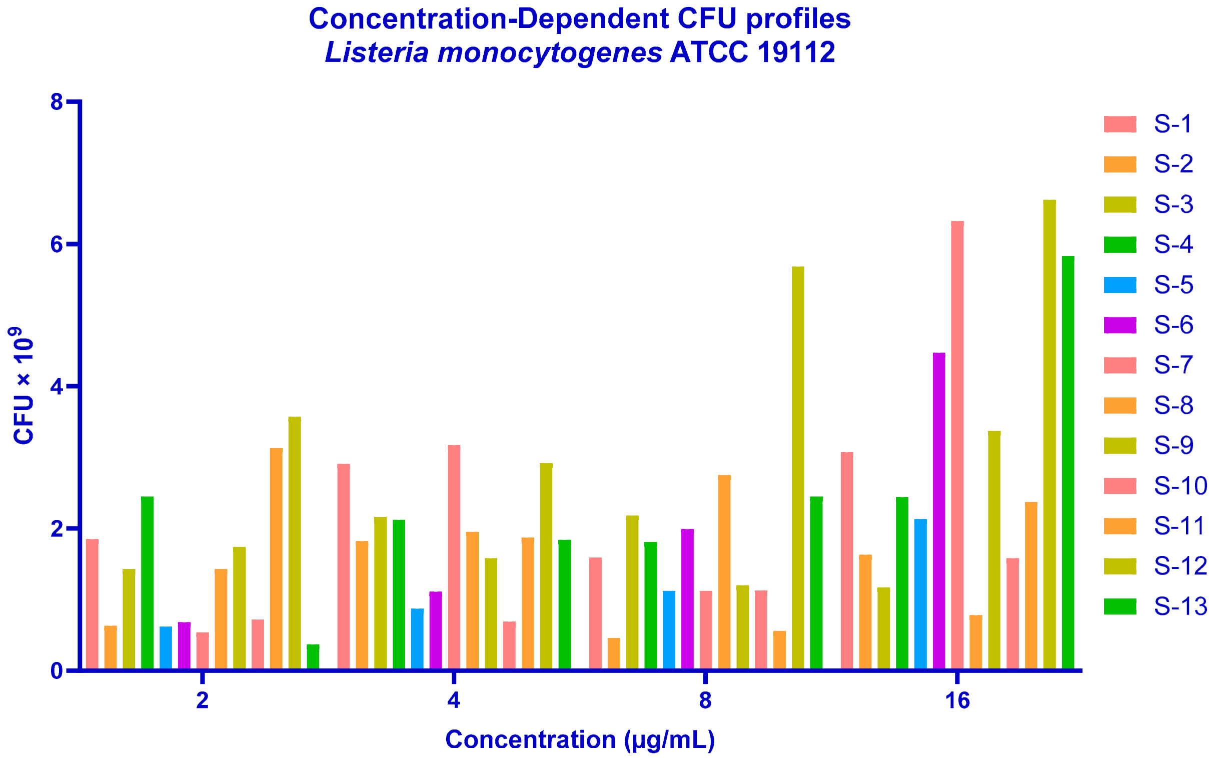

Concentration-Dependent CFU profiles
Listeria monocytogenes ATCC 19112
CFU × 10^9
8
6
4
2
0
2
4
8
16
Concentration (µg/mL)
S-1
S-2
S-3
S-4
S-5
S-6
S-7
S-8
S-9
S-10
S-11
S-12
S-13

Figure S1.F
